# Revisiting ring-degenerate rearrangements of 1-substituted-4-imino-1,2,3-triazoles

**DOI:** 10.3762/bjoc.14.184

**Published:** 2018-08-10

**Authors:** James T Fletcher, Matthew D Hanson, Joseph A Christensen, Eric M Villa

**Affiliations:** 1 Department of Chemistry, Creighton University, 2500 California Plaza, Omaha, NE 68178, U.S.A.

**Keywords:** colorimetric assay, condensation, imine exchange, rearrangement, 1,2,3-triazole

## Abstract

The 1-substituted-4-imino-1,2,3-triazole motif is an established component of coordination compounds and bioactive molecules, but depending on the substituent identity, it can be inherently unstable due to Dimroth rearrangements. This study examined parameters governing the ring-degenerate rearrangement reactions of 1-substituted-4-imino-1,2,3-triazoles, expanding on trends first observed by L’abbé et al. The efficiency of condensation between 4-formyltriazole and amine reactants as well as the propensity of imine products towards rearrangement was each strongly influenced by the substituent identity. It was observed that unsymmetrical condensation reactions conducted at 70 °C produced up to four imine products via a dynamic equilibrium of condensation, rearrangement and hydrolysis steps. Kinetic studies utilizing 1-(4-nitrophenyl)-1*H*-1,2,3-triazole-4-carbaldehyde with varying amines showed rearrangement rates sensitive to both steric and electronic factors. Such measurements were facilitated by a high throughput colorimetric assay to directly monitor the generation of a 4-nitroaniline byproduct.

## Introduction

Examples of multidentate chelators comprised of 1,2,3-triazole units have surged in recent years [1–7], enabled by efficient preparation from the Sharpless–Meldal copper-catalyzed azide–alkyne cycloaddition (CuAAC) reaction [8–11]. Click chelators with a variety of N-donor units connected at the 4-triazolyl position have been reported, including pyridine [12–17], quinoline [18–19], pyridazine [20–21], phthalazine [22], benzimidazole [23], phenanthroline [24], bipyridine [25] and amine [26] subunits. The utility of such chelators in constructing coordination compounds has been demonstrated for a wide range of transition metal and lanthanide cations, and have impacted fields that include bioimaging [26–27] and chemosensing [28–29].

Surprisingly, the reported examples partnering 1,2,3-triazole and imine N-donor groups are limited [30–32], despite the ubiquity of such Schiff base subunits in coordination chemistry. 4-Iminotriazole-containing molecules have also recently been shown to be useful synthons for preparing compounds with anticancer [33] and antituberculosis [34] properties. 1-Substituted-4-imino-1,2,3-triazole analogs are typically prepared from efficient condensation reactions of 1-substituted-4-formyl-1,2,3-triazoles deriving from CuAAC preparations [33,35–39]. Due to the thermodynamic stability of the imine bond at the 4-triazolyl position, this motif stands as an attractive target for designing new multidentate chelators to prepare coordination compounds.

**Figure 1:**
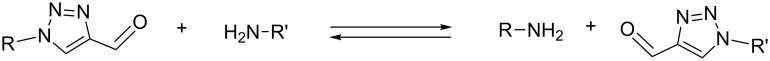
Conversion of 1-substituted-4-formyltriazole analogs via ring-degenerate rearrangement of 1-substituted-4-imino-1,2,3-triazoles as first described by L’abbé [42].

Cautiously, 1-substituted-4-imino-1,2,3-triazole analogs have been reported to display an inherent instability at elevated temperatures due to the propensity to undergo Dimroth rearrangements [40], as originally described by L’abbé et al. for this motif [41–43], as shown in Figure 1. This study demonstrated that such compounds are prone to rearrange by a ring-degenerate process promoted by an increasing electron-donor strength of the imine functionality. It was further demonstrated that this rearrangement could be utilized as a reliable synthetic tool, with 1-phenyl-1*H*-1,2,3-triazole-4-carbaldehyde being used to prepare 1-substituted-4-formyl-1,2,3-triazoles through a sequence of condensation, rearrangement and hydrolysis steps [42]. While this approach has found recent utility in preparing novel multidentate iminotriazole-based chelators [30–32], investigations exploring the parameters governing this rearrangement in greater detail than originally reported are lacking.

With an emerging interest in the 4-imino-1,2,3-triazole motif as coordination chemistry synthon, motivation for revisiting the ring degenerate rearrangements first described by L’abbe is twofold. A better understanding of parameters suppressing this rearrangement would inform the design of stable 4-imino-1,2,3-triazole target compounds, while a better understanding of parameters promoting this rearrangement might lead to improved methodology for the preparation of 1-subsituted-4-formyl-1,2,3-triazole target compounds. An expanded thermodynamic and kinetic investigation regarding the substituent effects of 4-imino-1,2,3-triazoles on this Dimroth rearrangement is therefore warranted, exemplary results of which are described herein.

## Results and Discussion

The original study of L’abbé regarding this ring-degenerate rearrangement focused on the condensation of 1-phenyl-1*H*-1,2,3-triazole-4-carbaldehyde with varying amines [42]. Rearrangement of the resulting 1-phenyl-4-imino-1,2,3-triazole derivatives was observed at 80 °C in both DMSO and alcohol solvents following the acid-catalyzed hydrolysis of iminotriazole product mixtures. An important goal of this current investigation was to expand the scope of this original study by also examining whether formyltriazole substituents influence the spontaneity of imine formation and subsequent susceptibility towards ring-degenerate rearrangement. To this end, a series of electron-withdrawing and electron-donating functional groups attached at the *para-*phenyl positions of both 1-phenyl-4-formyl-1,2,3-triazole and aniline reactants were used in the current investigation.

Two reaction conditions were employed in this study. Low temperature conditions used a 1:1 CHCl_3_/MeOH solvent system at room temperature, identified as useful for solubilizing the range of analogs included in the study and facilitating product work-up via simple evaporation. High-temperature conditions used a 1:1 *t*-BuOH/H_2_O solvent system at 70 °C, identified as useful in a previous study focusing on tandem CuAAC reaction development [35]. Importantly, instead of measuring only the relative amounts of formyltriazole products following imine hydrolysis promoted by reaction work-up, this study instead aimed to directly measure the relative amounts of both formyltriazole and iminotriazole product mixtures within each dynamic equilibrium.

Before examining the extent of rearrangement for unsymmetrical imine analogs, it was important to understand how the substituent identity might impact the spontaneity of imine formation in simplified systems not susceptible to productive rearrangement. Summarized in Table 1 is the observed extent of condensation for symmetrical pairings of aldehyde and imine reactants. Under both low and high-temperature conditions, the strongly electron-withdrawing nitro derivative was shown incapable of undergoing condensation. This can be attributed to the poor nucleophilicity of 4-nitroaniline. Condensation efficiency was observed to increase as the electron-donating character of the substituents increased. Hence, with the exception of 4-nitroaniline it was expected that each amine used in the study would be competent in forming appreciable quantities of imine product under both low and high-temperature conditions with unsymmetrical pairings of reactants.

**Table 1:** Evaluation of symmetrical condensation parameters.

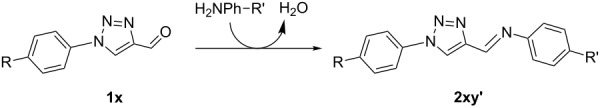								

entry	R	R’	solvent	conditions	compound	rel %^a^** 1x**	compound	rel %^a^** 2xy’**

1	NO_2_	NO_2_	MeOH/CHCl_3_	rt, 24 h	**1a**	100%	**2aa’**	0%
2	NO_2_	NO_2_	MeOH/CHCl_3_	rt, 48 h	**1a**	100%	**2aa’**	0%
3	NO_2_	NO_2_	MeOH/CHCl_3_	rt, 72 h	**1a**	100%	**2aa’**	0%
4	CF_3_	CF_3_	MeOH/CHCl_3_	rt, 24 h	**1b**	41%	**2bb’**	59%
5	CF_3_	CF_3_	MeOH/CHCl_3_	rt, 48 h	**1b**	17%	**2bb’**	83%
6	CF_3_	CF_3_	MeOH/CHCl_3_	rt, 72 h	**1b**	10%	**2bb’**	90%
7	H	H	MeOH/CHCl_3_	rt, 24 h	**1c**	0%	**2cc’**	100%
8	CH_3_	CH_3_	MeOH/CHCl_3_	rt, 24 h	**1d**	0%	**2dd’**	100%
9	OCH_3_	OCH_3_	MeOH/CHCl_3_	rt, 24 h	**1e**	0%	**2ee’**	100%
10	N(Et)_2_	N(Et)_2_	MeOH/CHCl_3_	rt, 24 h	**1f**	0%	**2ff’**	100%
11	NO_2_	NO_2_	*t*-BuOH/H_2_O	70 °C, 24 h	**1a**	100%	**2aa’**	0%
12	CF_3_	CF_3_	*t*-BuOH/H_2_O	70 °C, 24 h	**1b**	47%	**2bb’**	53%
13	H	H	*t*-BuOH/H_2_O	70 °C, 24 h	**1c**	11%	**2cc’**	89%
14	CH_3_	CH_3_	*t*-BuOH/H_2_O	70 °C, 24 h	**1d**	7%	**2dd’**	93%
15	OCH_3_	OCH_3_	*t*-BuOH/H_2_O	70 °C, 24 h	**1e**	2%	**2ee’**	98%
16	N(Et)_2_	N(Et)_2_	*t*-BuOH/H_2_O	70 °C, 24 h	**1f**	6%	**2ff’**	94%

^a^Relative molar fraction of **1** and **2** derivatives as determined by NMR integration.

**Figure 2:**
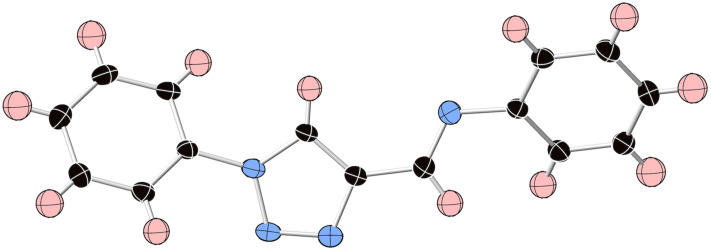
ORTEP structure of **2cc’**. Thermal ellipsoids shown at 25% probability.

Crystals of **2cc’** suitable for XRD analysis were grown from slow evaporation of a methylene chloride solution. As shown in Figure 2, each of the three aromatic rings in this molecule’s fully conjugated π-system adopts a largely coplanar configuration. The dihedral angle between the 1-phenyl substituent and the triazole ring is 28°, the dihedral angle between the imino group and triazole ring is 0.5° and the dihedral angle between the phenyl and imino group is 32°. This structural detail supports the ability of the 1-triazolyl and imino arene units to communicate electronically through the intervening triazole ring via largely coplanar π-systems within this family of compounds.

With the relative spontaneity of imine formation defined using symmetrical analogs, the study’s next focus was to examine how the substituent identity of both triazole and amine reactants influence the susceptibility of unsymmetrical imine analogs towards rearrangement. As summarized in Table 2, under condensation conditions this dynamic system can be expected to produce up to four unique imines and two unique aldehydes through a series of condensation, rearrangement and hydrolysis steps. The initial condensation reaction between formyltriazole and amine leads to iminotriazole formation (step a). This iminotriazole is then vulnerable to ring-degenerate Dimroth rearrangement (step b). This rearranged iminotriazole can then undergo hydrolysis to yield a new formyltriazole and a new amine byproduct (step c), each of which can then condense with their substituent-matched aldehyde analogs (steps d and e) to form their respective symmetrical iminotriazole products.

One expected outcome for this dynamic equilibrium was that step b would follow L’abbé’s previously observed rearrangement trend of being increasingly spontaneous for electron-rich amines partnered with electron-poor formyltriazoles [42]. Another expected outcome was that the condensation steps a, c and d would favor electron-rich amines relative to electron-poor amines, due to their superior nucleophilicity. All high-temperature reactions were analyzed after 24 h. (While this time point was not necessarily adequate for reaching chemical equilibrium within each dynamic system, it was deemed sufficient to make informative observations regarding the relative influence of substituent effects on product distributions.)

**Figure 3:**
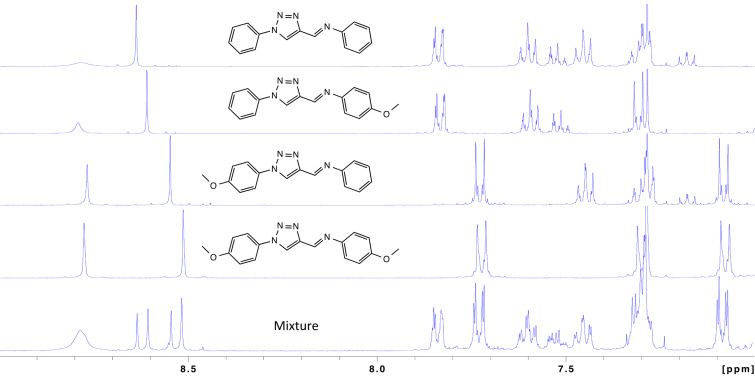
^1^H NMR aromatic region of a product mixture compared with reference imine analogs. Singlets appearing from 8.5–8.7 ppm correspond to the triazole C–H position used to quantify the imine constituents of product mixtures.

Direct, quantitative measurement of each triazole-containing species as a relative molar fraction within the dynamic reaction mixtures was made using ^1^H NMR analysis. This was accomplished by integration of the distinguishable triazole signals for each iminotriazole and formyltriazole analog contained in this study. Signal assignments were enabled by comparison with pure iminotriazoles prepared using low temperature conditions that promoted minimal rearrangement. An example of this comparative analysis is shown in Figure 3.

**Table 2:** Evaluation of unsymmetrical condensation parameters.

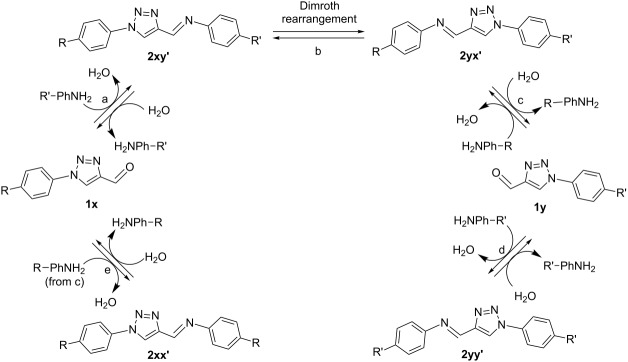														

entry	R	R’	compd	rel %^a^** 1x**	compd	rel %^a^** 2xy’**	compd	rel %^a^** 2yx’**	compd	rel %^a^** 1y**	compd	rel %^a^** 2yy’**	compd	rel %^a^** 2xx’**

1^b^	H	NO_2_	**1c**	100%	**2ca’**	0%	**2ac’**	0%	**1a**	0%	**2aa’**	0%	**2cc’**	0%
2^b^	H	CF_3_	**1c**	15%	**2cb’**	85%	**2bc’**	0%	**1b**	0%	**2bb’**	0%	**2cc’**	0%
3^b^	H	CH_3_	**1c**	3%	**2cd’**	97%	**2dc’**	0%	**1d**	0%	**2dd’**	0%	**2cc’**	0%
4^b^	H	OCH_3_	**1c**	0%	**2ce’**	100%	**2ec’**	0%	**1e**	0%	**2ee’**	0%	**2cc’**	0%
5^b^	H	N(Et)_2_	**1c**	8%	**2cf’**	92%	**2fc’**	0%	**1f**	0%	**2ff’**	0%	**2cc’**	0%
6^b^	NO_2_	H	**1a**	13%	**2ac’**	78%	**2ca’**	0%	**1d**	0%	**2cc’**	9%	**2aa’**	0%
7^b^	NO_2_	H 48 h	**1a**	18%	**2ac’**	57%	**2ca’**	0%	**1d**	0%	**2cc’**	25%	**2aa’**	0%
8^b^	NO_2_	H 72 h	**1a**	5%	**2ac’**	17%	**2ca’**	0%	**1d**	14%	**2cc’**	64%	**2aa’**	0%
9^b^	CF_3_	H	**1b**	41%	**2bc’**	59%	**2cb’**	0%	**1d**	0%	**2cc’**	0%	**2bb’**	0%
10^b^	CF_3_	H 48 h	**1b**	17%	**2bc’**	83%	**2cb’**	0%	**1d**	0%	**2cc’**	0%	**2bb’**	0%
11^b^	CF_3_	H 72 h	**1b**	10%	**2bc’**	90%	**2cb’**	0%	**1d**	0%	**2cc’**	0%	**2bb’**	0%
12^b^	CH_3_	H	**1d**	0%	**2dc’**	100%	**2cd’**	0%	**1d**	0%	**2cc’**	0%	**2dd’**	0%
13^b^	OCH_3_	H	**1e**	0%	**2ec’**	100%	**2ce’**	0%	**1d**	0%	**2cc’**	0%	**2ee’**	0%
14^b^	N(Et)_2_	H	**1f**	0%	**2fc’**	100%	**2cf’**	0%	**1d**	0%	**2cc’**	0%	**2ff’**	0%
15^c^	H	NO_2_	**1c**	100%	**2ca’**	0%	**2ac’**	0%	**1a**	0%	**2aa’**	0%	**2cc’**	0%
16^c^	H	CF_3_	**1c**	52%	**2cb’**	29%	**2bc’**	0%	**1b**	5%	**2bb’**	6%	**2cc’**	8%
17^c^	H	CH_3_	**1c**	0%	**2cd’**	31%	**2dc’**	22%	**1d**	0%	**2dd’**	28%	**2cc’**	19%
18^c^	H	OCH_3_	**1c**	2%	**2ce’**	24%	**2ec’**	23%	**1e**	2%	**2ee’**	31%	**2cc’**	18%
19^c^	H	N(Et)_2_	**1c**	8%	**2cf’**	14%	**2fc’**	28%	**1f**	13%	**2ff’**	15%	**2cc’**	22%
20^c^	NO_2_	H	**1a**	0%	**2ac’**	0%	**2ca’**	0%	**1d**	62%	**2cc’**	38%	**2aa’**	0%
21^c^	CF_3_	H	**1b**	0%	**2bc’**	3%	**2cb’**	18%	**1d**	62%	**2cc’**	14%	**2bb’**	3%
22^c^	CH_3_	H	**1d**	0%	**2dc’**	60%	**2cd’**	16%	**1d**	0%	**2cc’**	11%	**2dd’**	13%
23^c^	OCH_3_	H	**1e**	0%	**2ec’**	56%	**2ce’**	8%	**1d**	0%	**2cc’**	18%	**2ee’**	18%
24^c^	N(Et)_2_	H	**1f**	0%	**2fc’**	87%	**2cf’**	0%	**1d**	1%	**2cc’**	12%	**2ff’**	0%

^a^Relative molar fraction of **1** and **2** derivatives as determined by NMR integration; ^b^1:1 MeOH/CHCl_3_ solvent, room temperature, 24 h unless otherwise noted; ^c^1:1 *t*-BuOH/H_2_O solvent, 70 °C, 24 h unless otherwise noted.

Using this analytical approach, the extent to which temperature and substituent pairings impacted the population of dynamic product mixtures was successfully defined. Under low temperature conditions, the rearrangement was largely suppressed. For 1-phenyl-1*H*-1,2,3-triazole-4-carbaldehyde, the condensation efficiency increased with increasingly electron-rich anilines, and no rearrangements were observed (Table 2, entries 1–5). Evaluation of the formyltriazole substituent identity on the rearrangement surprisingly showed that even at room temperature those analogs with electron-poor nitro functionality underwent slow but consistent rearrangement (Table 2, entries 6–8). In contrast, all other analogs examined showed stability towards rearrangement at room temperature (Table 2, entries 9–14). These observations fit the previously observed trend of rearrangement skewing towards population of the electron-rich group at the 1-position of the triazole, and should inform the design of 1-substituted-4-imino-1,2,3-triazole target compounds with prescribed room temperature stability.

In contrast, under high-temperature conditions significant rearrangement occurred in nearly all of the unsymmetrical systems studied. The majority of substituent combinations resulted in formation of all four possible imine products, with those analogs possessing electron-rich substituents being the highest populated (Table 2, entries 15–24). As expected, the extent of rearrangement increased as the electron-donating properties of the amine reactants increased relative to the triazole. Unexpectedly, even those combinations where an electron-rich triazole was paired with an electron-poor aniline generated significant populations of rearranged imine products (Table 2, entries 16, and 22–24). This stands as a cautionary observation informing the design of 1-substituted-4-imino-1,2,3-triazoles intended for exposure to high-temperature conditions, such as commonly used in preparing metal coordination compounds. While the pairing of electron-rich formyltriazoles with electron-poor amines slows the rate of rearrangement, it does not preclude the ability of such analogs to rearrange significantly into scrambled analogs.

Among the substituent pairings studied, greatly simplified reaction outcomes were observed for those with nitro substituents. Those trials employing nitroaniline as a reactant showed a lack of imine formation due to its poor nucleophilicity (Table 2, entries 1 and 15). Those trials employing nitro-functionalized formyltriazole **1a** showed enhanced populations of rearranged formyltriazole and iminotriazole products lacking nitro functionality (Table 2, entries 6–8, and 20). This can be attributed to a two-fold influence by the nitro substituent. As an electron-poor formyltriazole, **1a** promotes forward progress of the rearrangement. Additionally, the resulting rearranged imine undergoes an irreversible hydrolysis due to the poor nucleophilicity of the *para*-nitroaniline byproduct. Because of the simplified high-temperature reaction outcomes of **1a** relative to the other analogs studied, additional investigation of this compound was undertaken.

**Figure 4:**
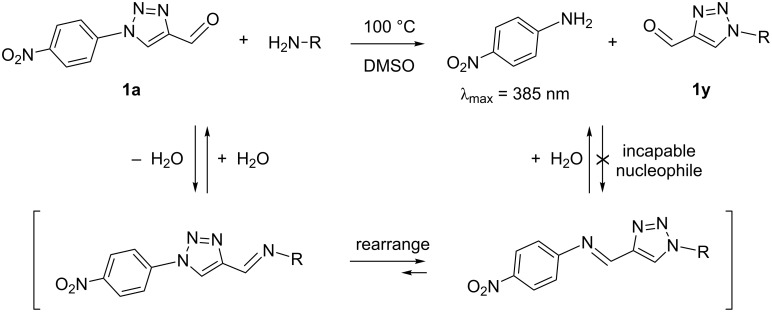
Rearrangement reaction progress of **1a** leading to irreversible formation of a colorimetric self-indicating byproduct.

With a secondary aim of identifying improved reaction conditions for exploiting this rearrangement as an efficient synthetic tool for formyltriazole preparations, it was proposed that **1a** might display significant advantages over previously utilized **1c** [30–32,42] as a synthon for this purpose. While **1c** showed scrambled substituents under the conditions studied when partnered with anilines with both electron-poor and electron-rich character, **1a** efficiently drove the reaction mixture towards rearranged, non-nitro products. It was hypothesized that in addition to promoting the forward rearrangement reaction and subsequent imine hydrolysis due to its nucleophilic incompetency, the *para*-nitroaniline byproduct formed in the condensation/rearrangement/hydrolysis might also serve as a colorimetric self-indicator of reaction progress, as illustrated in Figure 4.

In order to test the proposed advantages for using **1a** in preparing formyltriazole products, while also examining how amine identity impacts rearrangement rates, a high-throughput colorimetric assay exploiting the electronic properties of the *para*-nitroaniline byproduct was developed. DMSO was selected as the reaction solvent for these studies due to its proven utility in facilitating this reaction [42] and its low volatility. A reaction temperature of 100 °C was identified as useful for promoting significant reaction progress within a 24 h time window for the amine reactants studied. The increase in absorbance at 385 nm over time was used to calculate initial reaction rates for each of the amines studied, as described in the experimental section. It is important to note that this monitoring detects only the endpoint of the three-step sequence of condensation, rearrangement and hydrolysis steps, whereupon *para*-nitroaniline is released as a byproduct, and not each step individually.

As summarized in Table 3, it was observed that reaction rates were influenced by both electronic and steric properties of the amine reactant. A comparison of anilines showed that reaction rates decreased with increasing steric bulk at the *ortho* positions (Table 3, entries 1–4) and reaction rates increased with increasing electron-donating character of *para* substituents (Table 3, entries 5–7). Reaction rates also increased as the size (and corresponding polarizability) of aromatic amines increased (Table 3, entries 8–10). Similar steric trends were observed for aliphatic amines (Table 3, entries 11–13). ^1^H NMR analyses of selected product mixtures were used to verify that colorimetric responses correlated with the formation of formyltriazole rearrangement products and not from unexpected decomposition pathways. Demonstrating **1a** to be a useful synthon for formyltriazole preparations, it was used to prepare analogs **1c** (59% isolated yield) and **1d** (64% isolated yield) by the kinetic assay conditions (see Supporting Information File 1). Optimizing of reaction yields and exploring of the scope of amine reagents tolerated by this rearrangement reaction will be the subject of future studies.

**Table 3:** Kinetic assay results.^a^

entry	amine reactant	initial rate (μM/min)

1	2,6-dimethylaniline	20
2	*ortho*-toluidine	54
3	*meta*-toluidine	83
4	3,5-dimethylaniline	110
5	4-aminobenzotrifluoride	11
6	4-fluoroaniline	60
7	*para*-toluidine	67
8	2-naphthylamine	46
9	2-aminofluorene	91
10	2-aminoanthracene	130
11	3-aminopentane	30
12	*n*-hexylamine	91
13	benzylamine	120

^a^See experimental section in Supporting Information File 1.

## Conclusion

Motivated by an interest in the 4-imino-1,2,3-triazole synthon, the goal of this investigation was to better understand the susceptibility of such molecules to the ring-degenerate rearrangements first described by L’abbé in 1990 [42]. By directly measuring complex mixtures of aldehyde and imine products resulting from a dynamic combination of condensation, rearrangement and hydrolysis reactions between 1-substituted-4-formyl-1,2,3-triazoles and aromatic amines, additional insight regarding substituent effects on this process was defined. Importantly, while the established trend of relatively electron-rich amine reactants driving rearrangement of relatively electron-poor triazoles was verified, complex mixtures of scrambled 1-substituted-4-imino-1,2,3-triazole analogs were observed for nearly all combinations of reactants examined under elevated temperature conditions. Surprisingly, triazole analogs with electron-withdrawing substituents underwent significant rearrangement even at room temperature.

The spontaneous reactivity of 1-(4-nitrophenyl)-1*H*-1,2,3-triazole-4-carbaldehyde towards Dimroth rearrangement and its generation of a colorimetric indicator byproduct encouraged its use in a reaction kinetics investigation, where both electronic and steric properties of the amine reactant were shown to influence rearrangement rates. Having demonstrated its utility in studying this reaction using a high-throughput assay, it is proposed that 1-(4-nitrophenyl)-1*H*-1,2,3-triazole-4-carbaldehyde may stand as a synthon of improved practical utility for preparing 1-substituted-4-formyl-1,2,3-triazole target compounds via this ring-degenerate rearrangement process. Such will be the target of future studies.

## Supporting Information

Crystallographic data for the structures in this paper have been deposited with the Cambridge Crystallographic Data Centre: 2cc’ as CCDC 1842384. Copies of the data can be obtained, free of charge, on application to CCDC, 12 Union Road, Cambridge, CB2 1EZ, UK (fax: +44-(0)1223-336033 or e-mail: despoit@ccdc.cam.ac.uk).

File 1 Experimental procedures, copies ^1^H and ^13^C NMR spectra for all reported compounds, details of XRD analysis and UV–visible spectra for kinetic assays.
