# Supplementary material for: Revisiting ring-degenerate rearrangements of 1-substituted-4-imino-1,2,3-triazoles
Source: Beilstein J Org Chem. 2018 Aug 10;14:2098–105. doi: 10.3762/bjoc.14.184 (PMC6122373; doi:10.3762/bjoc.14.184)
Supplement: File 1 — Experimental procedures, copies 1H and 13C NMR spectra for all reported compounds, details of XRD analysis and UV–visible spectra for kinetic assays. [file Beilstein_J_Org_Chem-14-2098-s001.pdf]

Supporting Information

for

Revisiting ring-degenerate rearrangements of 1-substituted-4-imino-1,2,3-triazoles

James T. Fletcher*, Matthew D. Hanson, Joseph A. Christensen and Eric M. Villa

Address: Department of Chemistry, Creighton University, 2500 California Plaza, Omaha, NE 68178, U.S.A.

Email: James T. Fletcher - jamesfletcher@creighton.edu

*Corresponding author

Experimental procedures, copies 1H and 13C NMR spectra for all reported compounds, details of XRD analysis and UV-visible spectra for kinetic assays

Table of Contents:

Page	
S2–S8	Experimental procedures
S9–10	^{1}H and ^{13}C NMR spectra of **2cc'**
S11–12	^{1}H and ^{13}C NMR spectra of **2dd'**
S13–14	^{1}H and ^{13}C NMR spectra of **2ee'**
S15–16	^{1}H and ^{13}C NMR spectra of **2ff'**
S17–18	^{1}H and ^{13}C NMR spectra of **2dc'**
S19–20	^{1}H and ^{13}C NMR spectra of **2ec'**
S21–22	^{1}H and ^{13}C NMR spectra of **2fc'**
S23–24	^{1}H and ^{13}C NMR spectra of **2cd'**
S25–26	^{1}H and ^{13}C NMR spectra of **2ce'**
S27–28	UV–vis data for kinetic studies

Experimental procedures

General experimental details

Aldehydes **1a**, **1b**, **1c**, **1d**, **1e** and **1f** were prepared as previously reported [1]. *N*,*N*-diethyl-*p*-phenylenediamine was used as purchased from Sigma-Aldrich. 4-Aminobenzotrifluoride and aniline were used as purchased from Oakwood Chemical. 4-Nitroaniline, *p*-toluidine and *p*-anisidine were used as purchased from Acros. Solvents were used as purchased from Fisher Scientific. Deuterated solvents for NMR analysis were used as purchased from Cambridge Isotopes. NMR analyses were obtained on a 400 MHz Bruker Ascend system. HRMS analyses were acquired on a Bruker micrOTOF-Q III system. Attempts to promote $[M + H]^+$ ions using 0.1% formic acid in acetonitrile resulted in degradation of imine functionality in the spectrometer. The use of neat acetonitrile reliably produced observable $[M + Na]^+$ ions. Melting points were acquired using a Stanford Research Systems Optimelt apparatus. Crystallographic data for the structure in this paper has been deposited with the Cambridge Crystallographic Data Centre as 1842384. Copies of the data can be obtained, free of charge, on application to CCDC, 12 Union Road, Cambridge, CB2 1EZ, UK (fax: +44-(0)1223-336033 or e-mail: despoit@ccdc.cam.ac.uk).

General procedure for condensation reactions at low temperature

Aldehyde (0.1 mmol) and amine (0.1 mmol) reactants were dissolved in 2.5 mL methanol and 2.5 mL chloroform and stirred at room temperature in a sealed vial. Products were isolated by air evaporation of solvent at room temperature.

N-[(1-Phenyl-1H-1,2,3-triazol-4-yl)methylene]benzenamine (2cc')

Colorless crystalline solid, 85% yield, mp 124-126 °C. ^{1}H NMR (400 MHz, $CDCl_3$): δ 8.75 (s, 1H), 8.61 (s, 1H), 7.81 (m, 2H), 7.57 (m, 2H), 7.51 (m, 1H), 7.43 (m, 2H), 7.27 (m, 3H); ^{13}C NMR (400 MHz, $CDCl_3$): δ 152.0, 151.3, 147.7, 136.9, 130.1, 129.51, 129.48, 126.9, 121.2, 121.1, 120.9; HRMS (ESI) *m/z*: Calcd for $C_{15}H_{12}N_4Na$ $[M+Na]^+$ 271.0954, found 271.0944.

4-Methyl-N-[[1-(4-methylphenyl)-1H-1,2,3-triazol-4-yl]methylene]benzenamine (2dd')

Colorless crystalline solid, 89% yield, mp 165-166 °C. ^{1}H NMR (400 MHz, $CDCl_3$): δ 8.75 (s, 1H), 8.55 (s, 1H), 7.67 (d, J = 8.4 Hz, 2H), 7.35 (d, J = 8.4 Hz, 2H), 7.23 (d, J = 8.4 Hz, 2H), 7.18 (d, J = 8.4 Hz, 2H), 2.44 (s, 3H), 2.38 (s, 3H); ^{13}C NMR (400 MHz, $CDCl_3$): δ 151.2, 148.7, 147.7, 139.6, 136.8, 134.6, 130.6, 130.1, 121.02, 120.98, 120.7, 21.3, 21.2; HRMS (ESI) *m/z*: Calcd for $C_{17}H_{16}N_4Na$ $[M+Na]^+$ 299.1267, found 299.1253.

4-Methoxy-N-[[1-(4-methoxyphenyl)-1H-1,2,3-triazol-4-yl]methylene]benzenamine (2ee')

Colorless crystalline solid, 91% yield, mp 149-150 °C. ^{1}H NMR (400 MHz, $CDCl_3$): δ 8.75 (s, 1H), 8.49 (s, 1H), 7.71 (d, J = 8.8 Hz, 2H), 7.27 (d, J = 8.8 Hz, 2H), 7.05 (d, J = 8.8 Hz, 2H), 6.95 (d, J = 8.8 Hz, 2H), 3.89 (s, 3H), 3.84 (s, 3H); ^{13}C NMR (400 MHz, $CDCl_3$): δ 160.4, 159.0, 149.9, 147.8, 144.2, 130.3, 122.5, 122.4, 120.9, 115.1, 114.7, 55.9, 55.7; HRMS (ESI) *m/z*: Calcd for $C_{17}H_{16}N_4O_2Na$ $[M+Na]^+$ 331.1165, found 331.1172.

4-Diethylamino-*N*-[[1-[4-(diethylamino)phenyl]-1*H*-1,2,3-triazol-4-yl]methylene]benzenamine (2ff')

Black powder, 88% yield, mp 145 °C (decomp). ^{1}H NMR (400 MHz, $CDCl_3$): δ 8.77 (s, 1H), 8.42 (s, 1H), 7.56 (d, *J* = 9.2 Hz, 2H), 7.28 (d, *J* = 9.2 Hz, 2H), 6.73 (d, *J* = 9.2 Hz, 2H), 6.70 (d, *J* = 9.2 Hz, 2H), 3.41 (q, *J* = 7.2 Hz, 4H), 3.39 (q, *J* = 7.2 Hz, 4H), 1.21 (t, *J* = 7.1 Hz, 6H), 1.19 (t, *J* = 7.2 Hz, 6H); ^{13}C NMR (400 MHz, $CDCl_3$): δ 148.4, 147.9, 147.4, 146.9, 139.1, 125.7, 122.8, 122.4, 120.1, 112.2, 111.8, 44.8, 44.7, 12.8, 12.7; HRMS (ESI) *m/z*: Calcd for $C_{23}H_{30}N_6Na$ $[M+Na]^+$ 413.2424, found 413.2413.

***N*-[[1-(4-Methylphenyl)-1*H*-1,2,3-triazol-4-yl]methylene]benzenamine (2dc')**

Colorless crystalline solid, 84% yield, mp 159-160 °C. ^{1}H NMR (400 MHz, $CDCl_3$): δ 8.75 (s, 1H), 8.56 (s, 1H), 7.68 (d, *J* = 8.4 Hz, 2H), 7.42 (m, 2H), 7.36 (d, *J* = 8.4 Hz, 2H), 7.27 (m, 3H), 2.45 (s, 3H); ^{13}C NMR (400 MHz, $CDCl_3$): δ 152.1, 151.3, 147.6, 139.7 134.6, 130.6, 129.5, 126.8, 121.2, 121.1, 120.8, 21.3; HRMS (ESI) *m/z*: Calcd for $C_{16}H_{14}N_4Na$ $[M+Na]^+$ 285.1111, found 285.1105.

***N*-[[1-(4-Methoxyphenyl)-1*H*-1,2,3-triazol-4-yl]methylene]benzenamine (2ec')**

Tan crystalline solid, 83% yield, mp 115 °C (decomp). ^{1}H NMR (400 MHz, $CDCl_3$): δ 8.74 (s, 1H), 8.52 (s, 1H), 7.70 (d, *J* = 9.2 Hz, 2H), 7.42 (m, 2H), 7.26 (m, 3H), 7.06 (d, *J* = 9.2 Hz, 2H), 3.89 (s, 3H); ^{13}C NMR (400 MHz, $CDCl_3$): δ 160.4, 152.2, 151.3, 147.5, 130.3, 129.5, 126.8, 122.5, 121.2, 121.1, 115.2, 55.9; HRMS (ESI) *m/z*: Calcd for $C_{16}H_{14}N_4ONa$ $[M+Na]^+$ 301.1060, found 301.1075.

N-[[1-[4-(Diethylamino)phenyl]-1H-1,2,3-triazol-4-yl]methylene]benzenamine (2fc')

Dark brown crystalline solid, 89% yield, mp 131 °C (decomp). ^{1}H NMR (400 MHz, $CDCl_3$): δ 8.74 (s, 1H), 8.46 (s, 1H), 7.57 (d, *J* = 9.2 Hz, 2H), 7.42 (m, 2H), 7.26 (m, 3H), 6.74 (d, *J* = 9.2 Hz, 2H), 3.42 (q, *J* = 7.2 Hz, 2H), 1.21 (t, *J* = 7.2 Hz, 3H); ^{13}C NMR (400 MHz, $CDCl_3$): δ 152.5, 151.5, 148.5, 147.2, 129.5, 126.7, 125.5, 122.4, 121.1, 120.9, 111.8, 44.8, 12.7; HRMS (ESI) *m/z*: Calcd for $C_{19}H_{21}N_5Na$ $[M+Na]^+$ 342.1689, found 342.1697.

4-Methyl-N-[(1-phenyl-1H-1,2,3-triazol-4-yl)methylene]benzenamine (2cd')

Colorless crystalline solid, 92% yield, mp 112-113 °C. ^{1}H NMR (400 MHz, $CDCl_3$): δ 8.76 (s, 1H), 8.60 (s, 1H), 7.81 (m, 2H), 7.57 (m, 2H), 7.50 (m, 1H), 7.23 (d, *J* = 8.4 Hz, 2H), 7.19 (d, *J* = 8.4 Hz, 2H), 2.39 (s, 3H); ^{13}C NMR (400 MHz, $CDCl_3$): δ 150.9, 148.5, 147.7, 136.7, 130.4, 130.0, 129.3, 120.9, 120.8, 120.7, 120.5, 21.2; HRMS (ESI) *m/z*: Calcd for $C_{16}H_{14}N_4Na$ $[M+Na]^+$ 285.1111, found 285.1113.

4-Methoxy-N-[(1-phenyl-1H-1,2,3-triazol-4-yl)methylene]benzenamine (2ce')

Colorless crystalline solid, 90% yield, mp 125-126°C. ^{1}H NMR (400 MHz, $CDCl_3$): δ 8.76 (s, 1H), 8.58 (s, 1H), 7.80 (m, 2H), 7.56 (m, 2H), 7.49 (m, 1H), 7.28 (d, *J* = 8.8 Hz, 2H), 6.95 (d, *J* = 8.8 Hz, 2H), 3.85 (s, 3H); ^{13}C NMR (400 MHz, $CDCl_3$): δ 159.0, 149.7, 147.9, 144.1, 136.9, 130.1, 129.4, 122.5, 120.8, 115.1, 114.7, 55.7; HRMS (ESI) *m/z*: Calcd for $C_{16}H_{14}N_4ONa$ $[M+Na]^+$ 301.1060, found 301.1073.

General procedure for condensation reactions at high temperature

Aldehyde (0.1 mmol) and amine (0.1 mmol) reactants were dissolved in 2.5 mL water and 2.5 mL *tert*-butanol and stirred at 70 ^{o}C in a sealed vial. Products were isolated by extraction between methylene chloride and 5% NH_4OH (aq), organic layer separated and dried over $MgSO_4$, gravity filtered and solvent removed via rotary evaporation.

Kinetic assays

Aldehyde and amine reactants were mixed 1:1 in DMSO at 10 mM concentrations and heated to 100 ^{o}C. At desired timepoints, 2 μL aliquots were removed for each reaction and diluted 1:100 into DMSO in a 96 well plate at room temperature. Following the collection of the final timepoints, the absorbance of the plate was read using a plate reader. Reaction progress was monitored as an increase in the *para*-nitroaniline absorbance at 385 nm, which did not overlap with reactant or imine product absorbance. Control reactions showed that neither **1a** itself nor the amines studied decomposed under these conditions to give absorbance in this region. Absorbance values acquired during the initial phase of each reaction (60–90 min) were plotted using Excel and fit to a linear progression. The slope of each fit was used to calculate the initial rates of the overall rearrangement reactions using the values $\varepsilon = 13{,}500$ $M^{-1}cm^{-1}$; path length = 0.6 cm to covert ΔAbs(385nm)/min into Δ[μM]/min.

Preparation of 1c from 1a

Aniline (18 mL, 0.20 mmol) and **1a** (44 mg, 0.20 mmol) were dissolved in DMSO (5 mL) and stirred at 100 ^{o}C for 24 h. Upon cooling, the reaction was diluted into diethyl ether and washed 3x with 6 M HCl. The ether layer was dried over $MgSO_4$ and gravity filtered.

Volatiles were removed via rotary evaporation to give **1c** (23 mg, 0.12 mmol, 59%). Characterization matched that previously reported.[1]

Preparation of 1d from 1a

4-Toluidine (21 mg, 0.20 mmol) and **1a** (44 mg, 0.20 mmol) were dissolved in DMSO (5 ml) and stirred at 100 ^{o}C for 24 h. Upon cooling, the reaction was diluted into diethyl ether and washed 3× with 6 M HCl. The ether layer was dried over $MgSO_4$ and gravity filtered. Volatiles were removed via rotary evaporation to give **1d** (28 mg, 0.13 mmol, 67%). Characterization matched that previously reported.[1]

XRD analysis

Refinement details: The crystal was mounted on MiTeGen Microloop with non-drying immersion oil and then optically aligned on the Rigaku SCX-Mini diffractometer using a digital camera. Initial matrix images were collected to determine the unit cell, validity and proper exposure time. Three hemispheres (where ϕ = 0.0, 120.0 and 240.0) of data were collected with each consisting 180 images each with 1.00° widths and a 1.00° step. CrysAlis PRO 1.171.38.46 [2] was used for integration, scaling and absorption correction. The structures were initially solved and then refined using SHELXT Intrinsic Phasing and SHELXL [3]. Olex2 [4] was used as a graphical interface. The image for the above compound was generated using CrystalMaker® version 9.2.8: a crystal and molecular structures program for Mac and Windows (CrystalMaker Software Ltd, Oxford, England, www.crystalmaker.com). The refinement proceeded without any incidents and without any need for modelling disorder or twinning or any constraints or restraints.

Crystal data for **2cc'**, $C_{15}H_{12}N_4$ (M = 248.29 g/mol): monoclinic, space group C2/c (no. 15), a = 18.071(2) Å, b = 5.7602(4) Å, c = 24.808(2) Å, β = 90.889(7)°, V = 2582.0(4) Å^3, Z = 8, T = 293(2) K, μ(Mo Kα) = 0.080 mm^{-1}, $Dcalc$ = 1.277 g/cm^3, 13315 reflections measured (3.284° ≤ 2Θ ≤ 56.558°), 3218 unique (R_{int} = 0.0432, R_{sigma} = 0.0367) which were used in all calculations. The final R_1 was 0.0492 (I > 2σ(I)) and wR_2 was 0.1079 (all data).

References

1. Fletcher, J.T.; Christensen, J.A.; Villa, E.M. *Tetrahedron Lett*. **2017**, *58*, 4450-4454.

2. Rigaku, O.D. *CrysAlis PRO. 1.171.38.43* . Rigaku Oxford Diffraction, Yarnton, England, 2015.

3. Sheldrick, G.M. *Acta Cryst. A*. **2015**, *71*, 3-8.

4. Palmer D, Conley M, Parsonson L, Rimmer L, *I. S. CrystalMaker*, 2017.

^{1}H NMR spectrum of **2cc'**

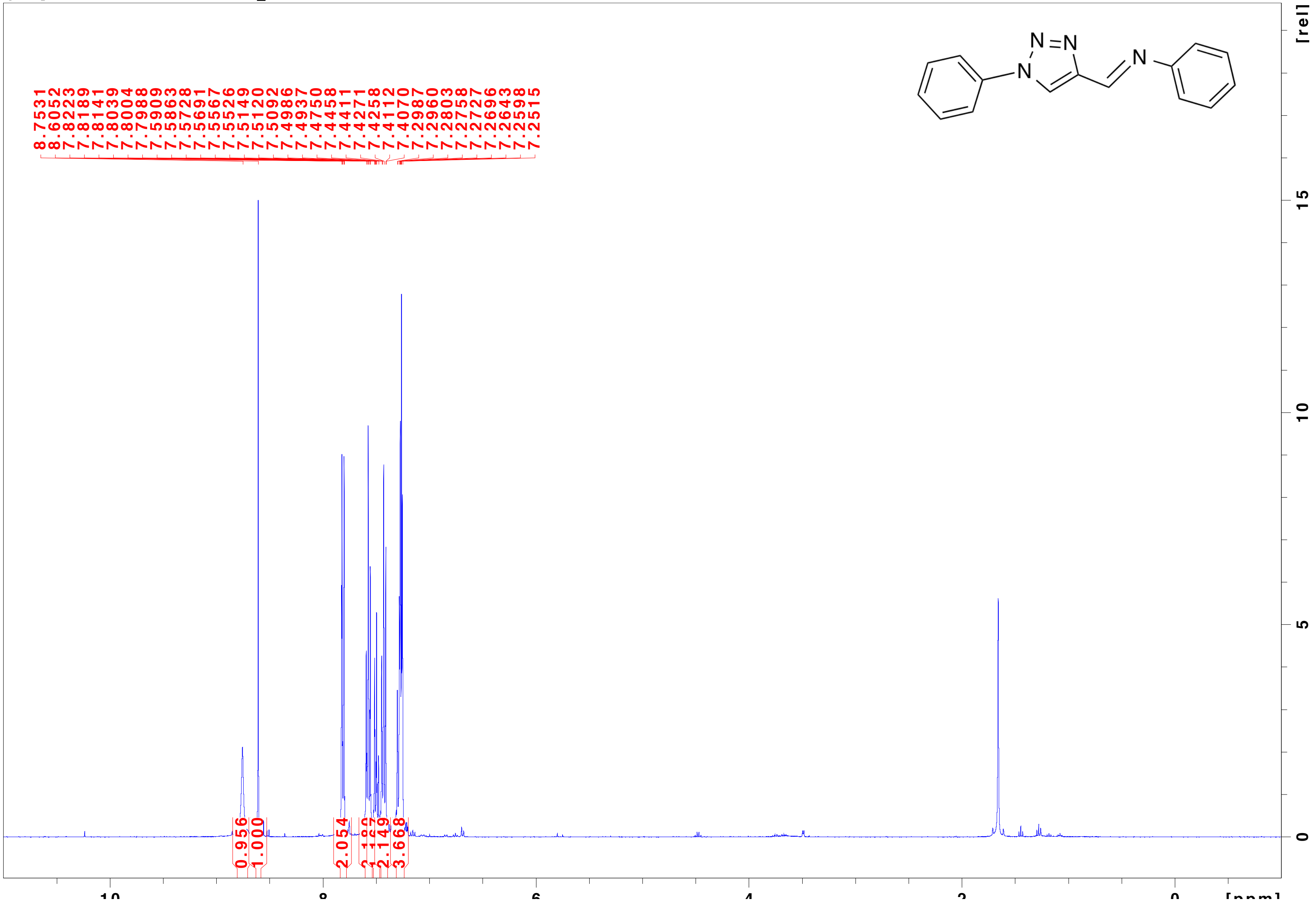

^{13}C NMR spectrum of **2cc’**

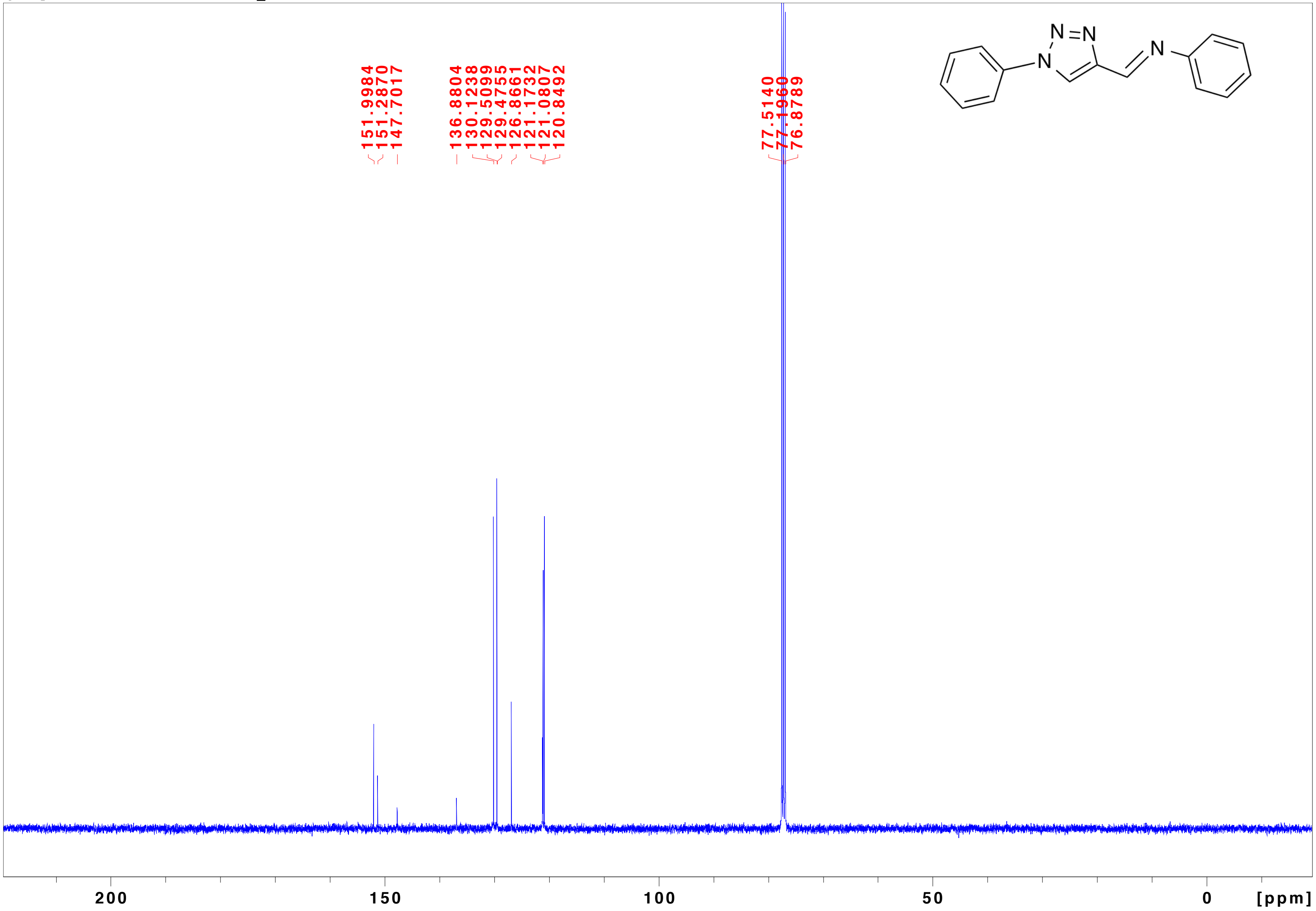

^{1}H NMR spectrum of **2dd'**

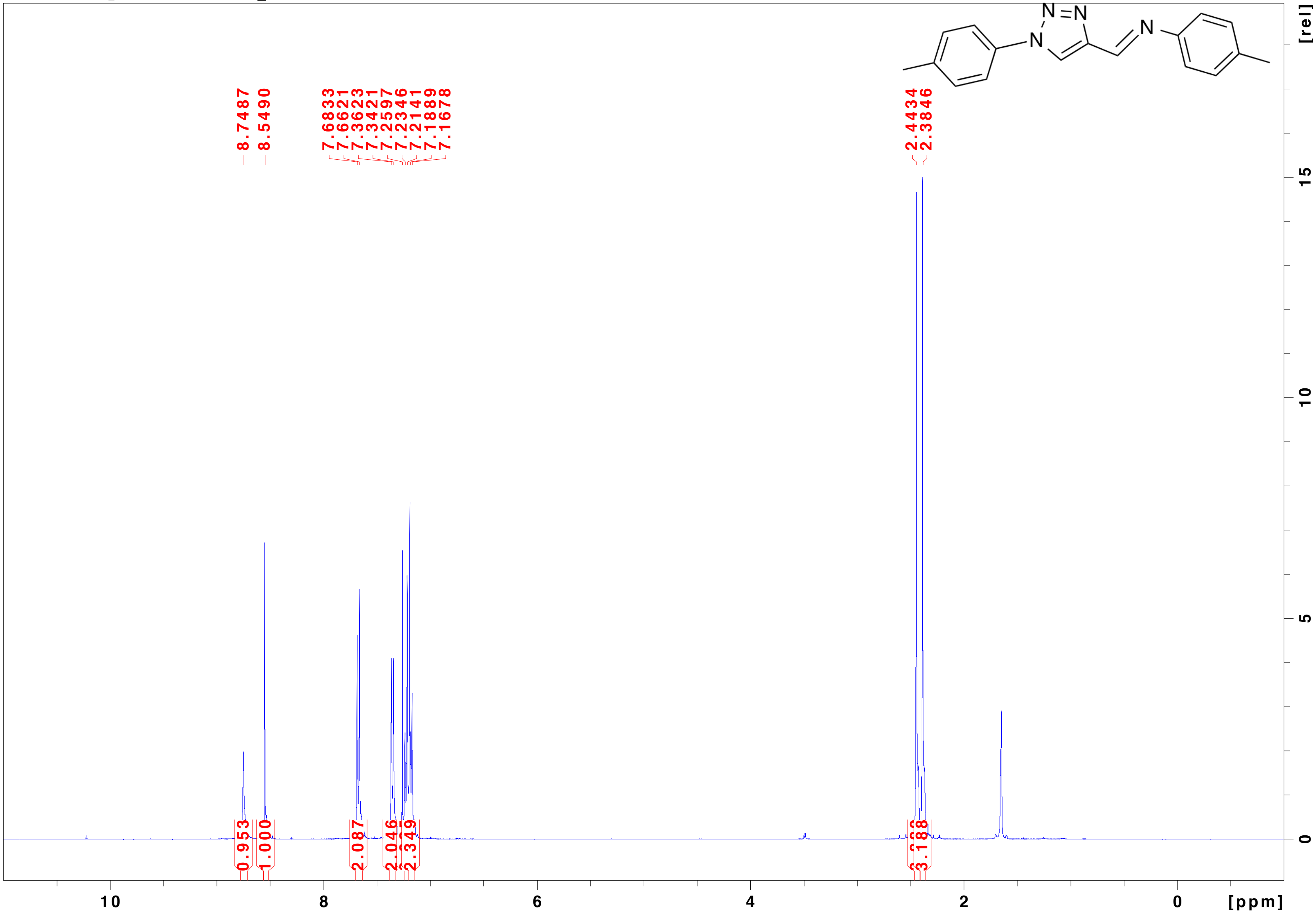

^{13}C NMR spectrum of **2dd'**

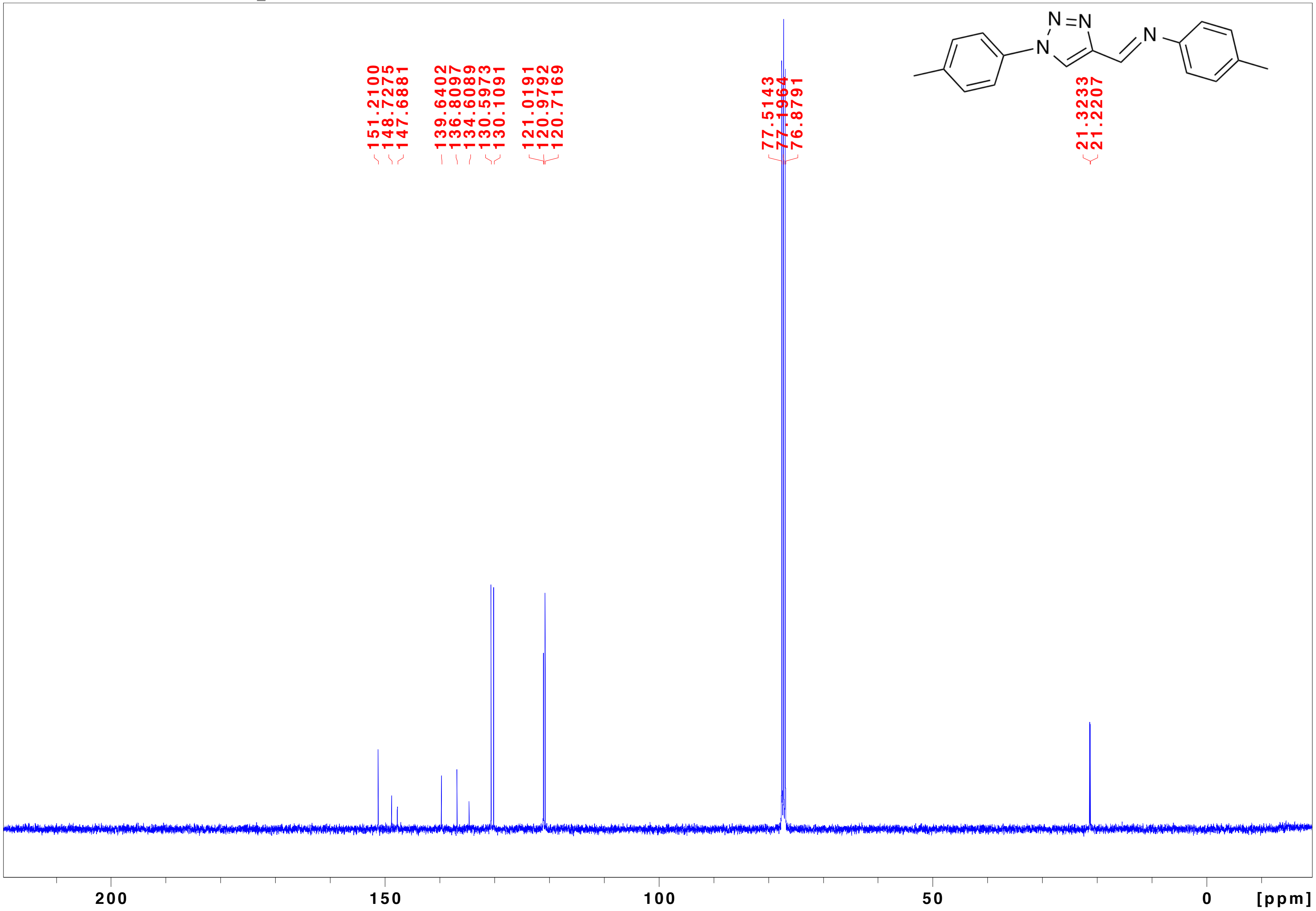

^{1}H NMR spectrum of **2ee'**

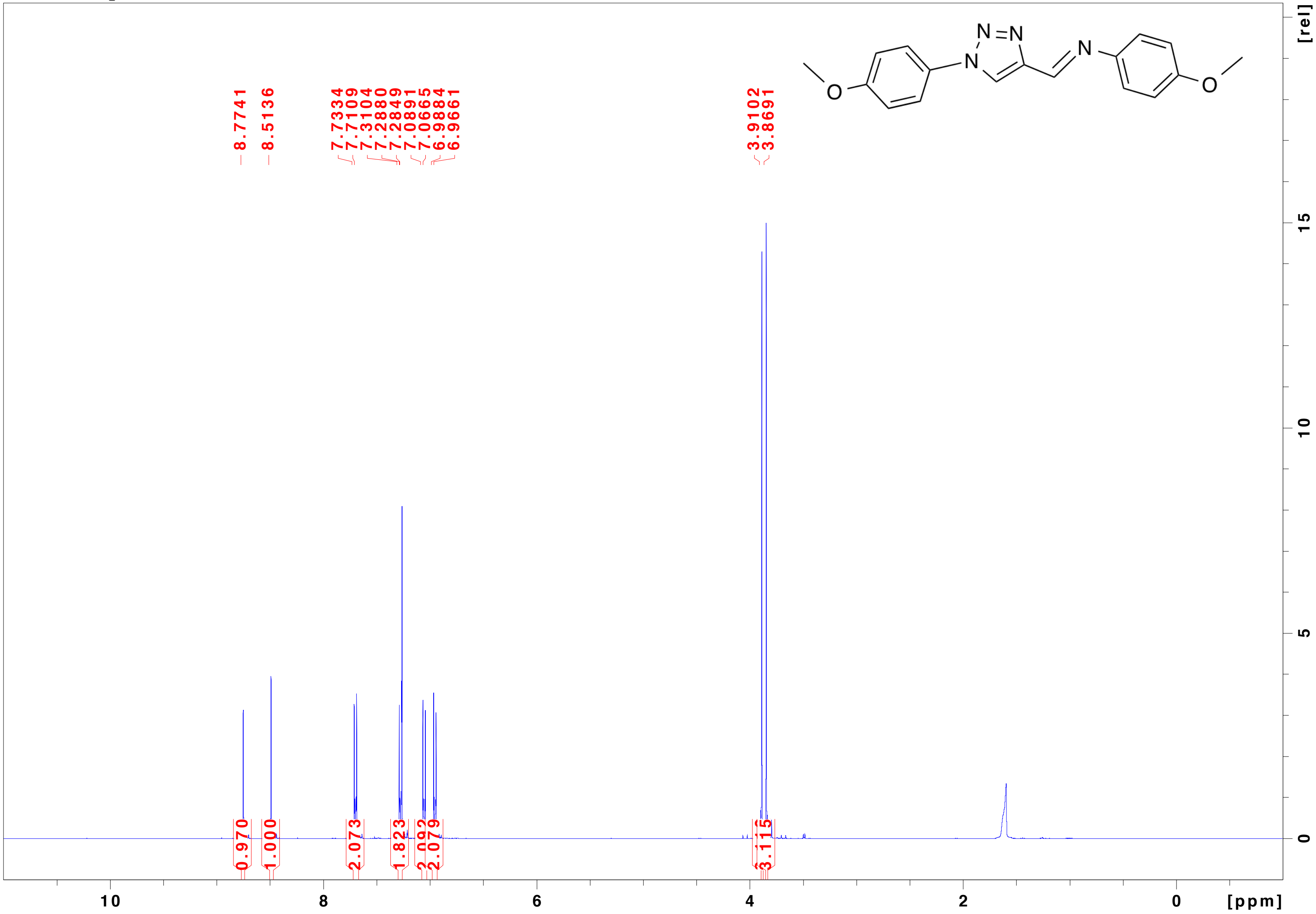

^{13}C NMR spectrum of **2ee'**

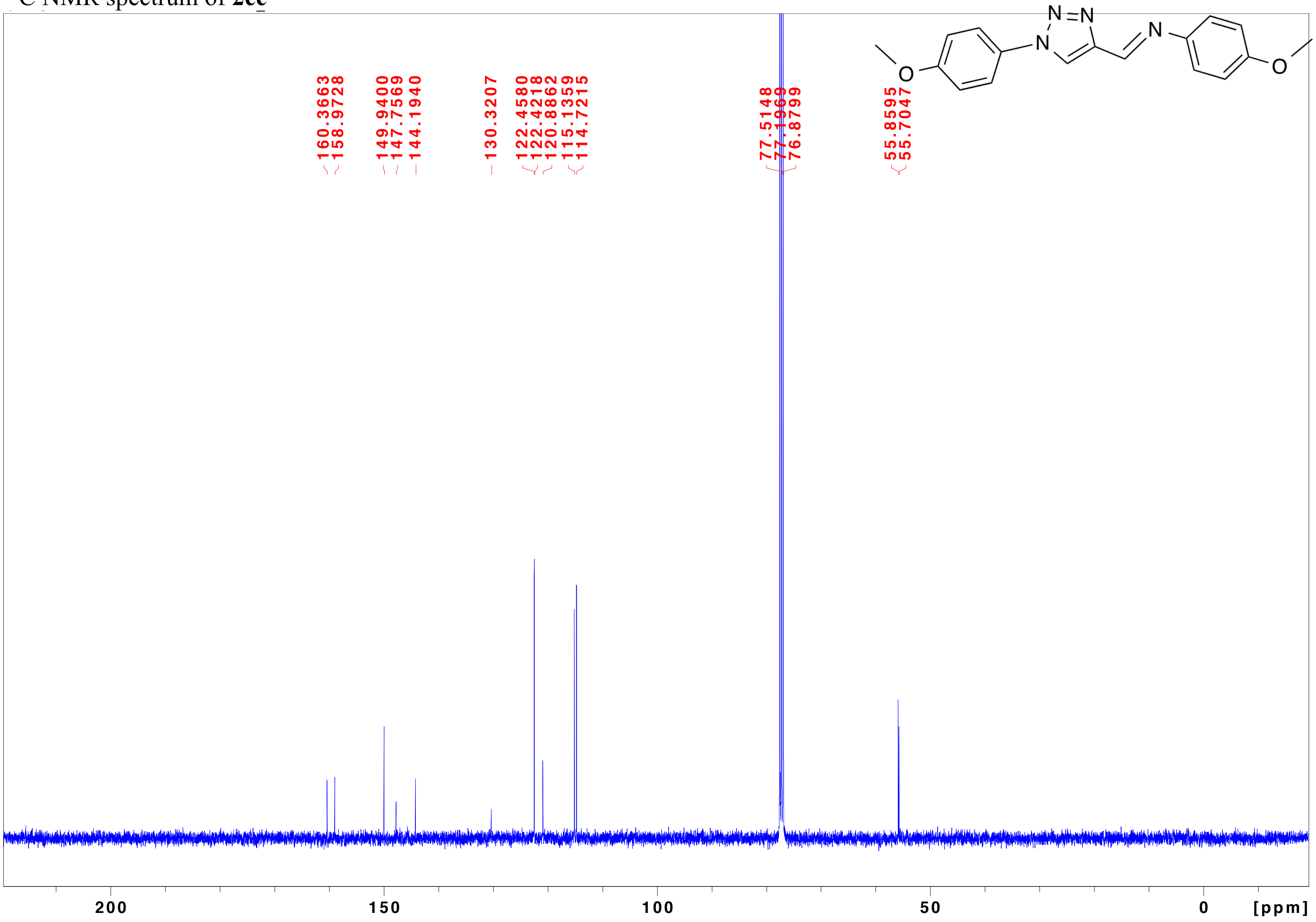

^{1}H NMR spectrum of **2ff'**

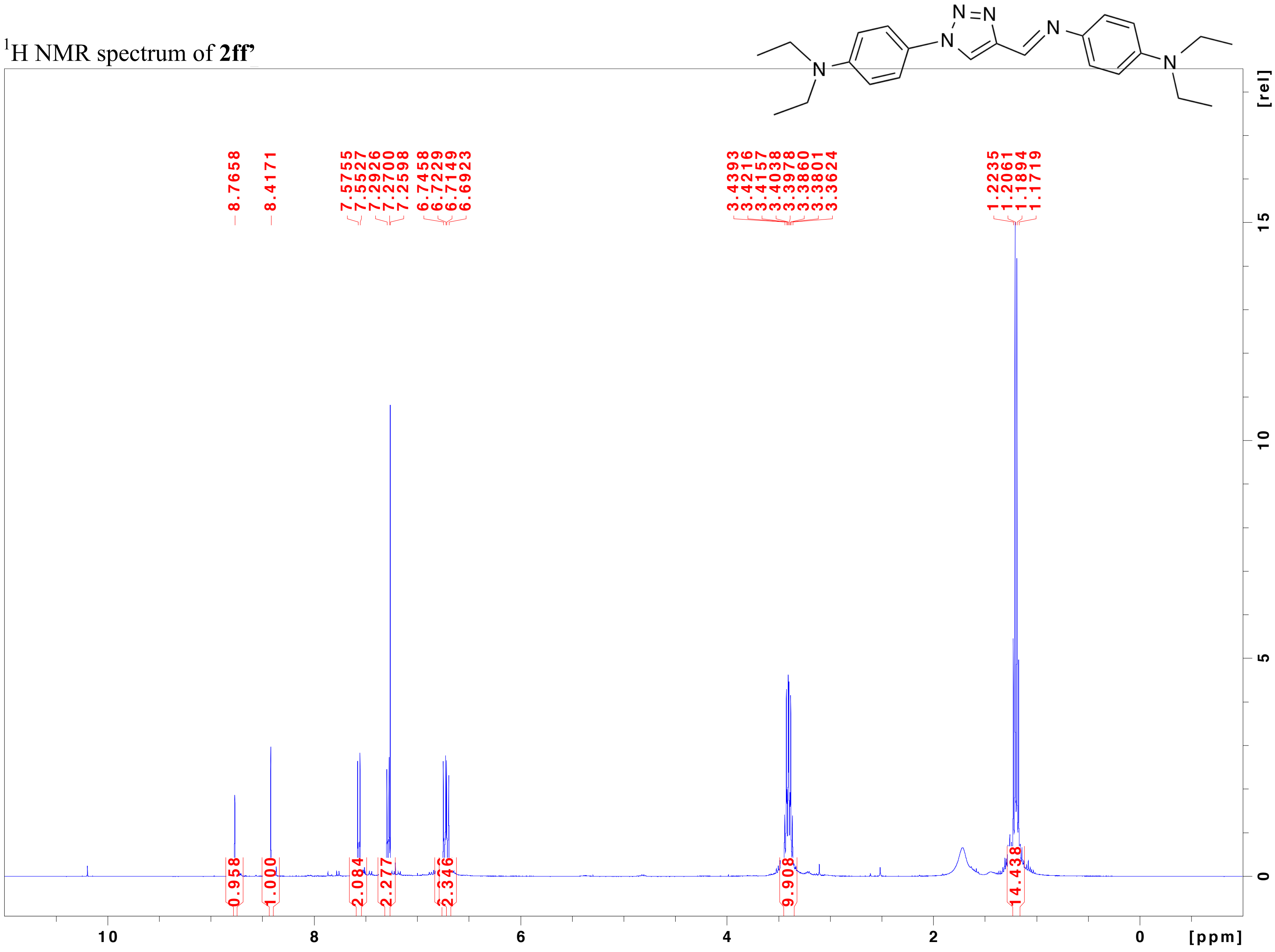

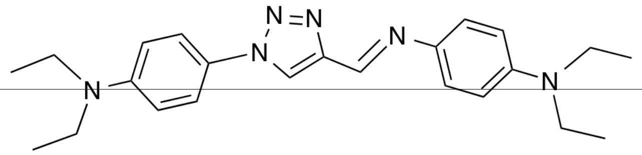

^{13}C NMR spectrum of **2ff'**

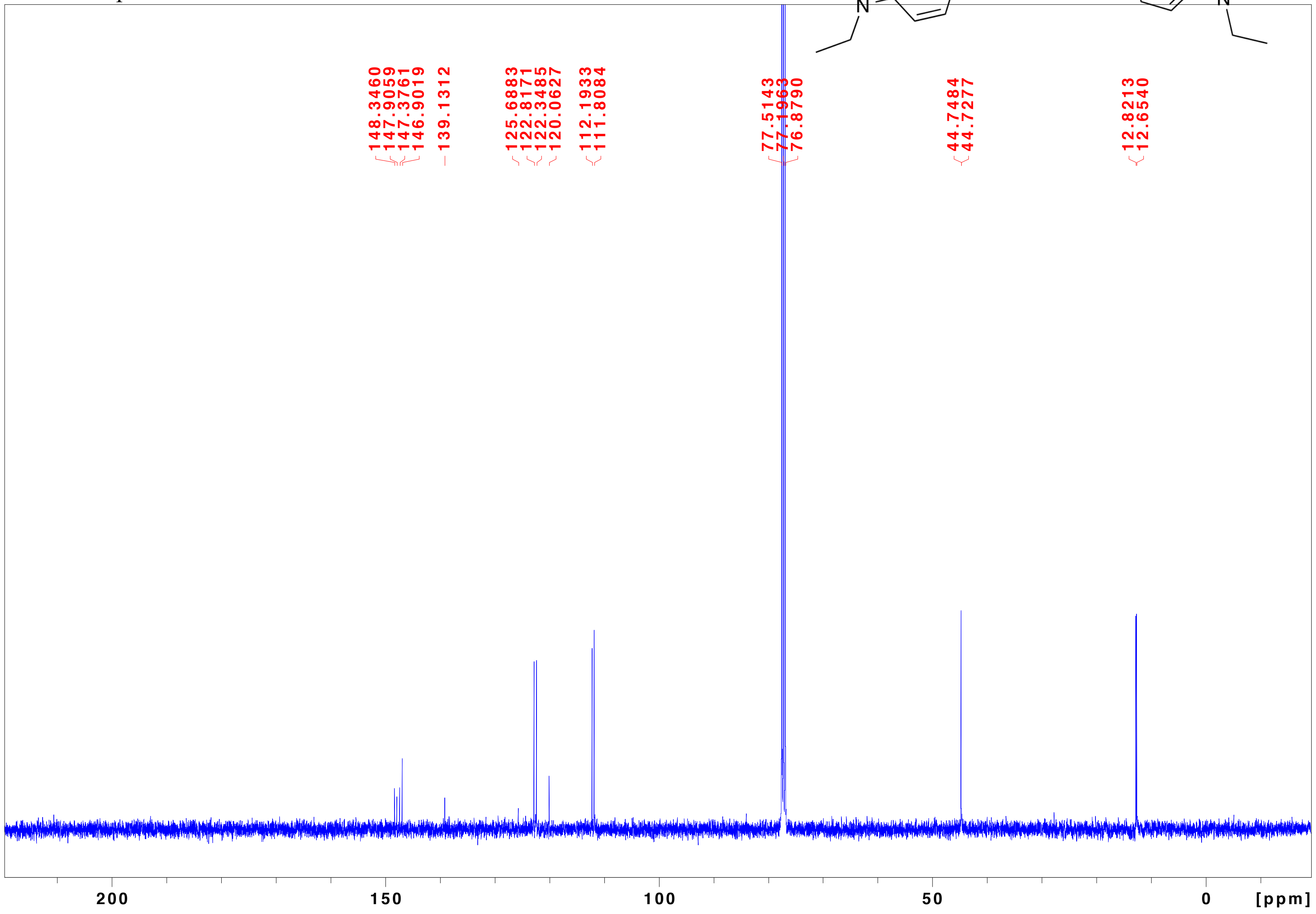

^{1}H NMR spectrum of **2dc’**

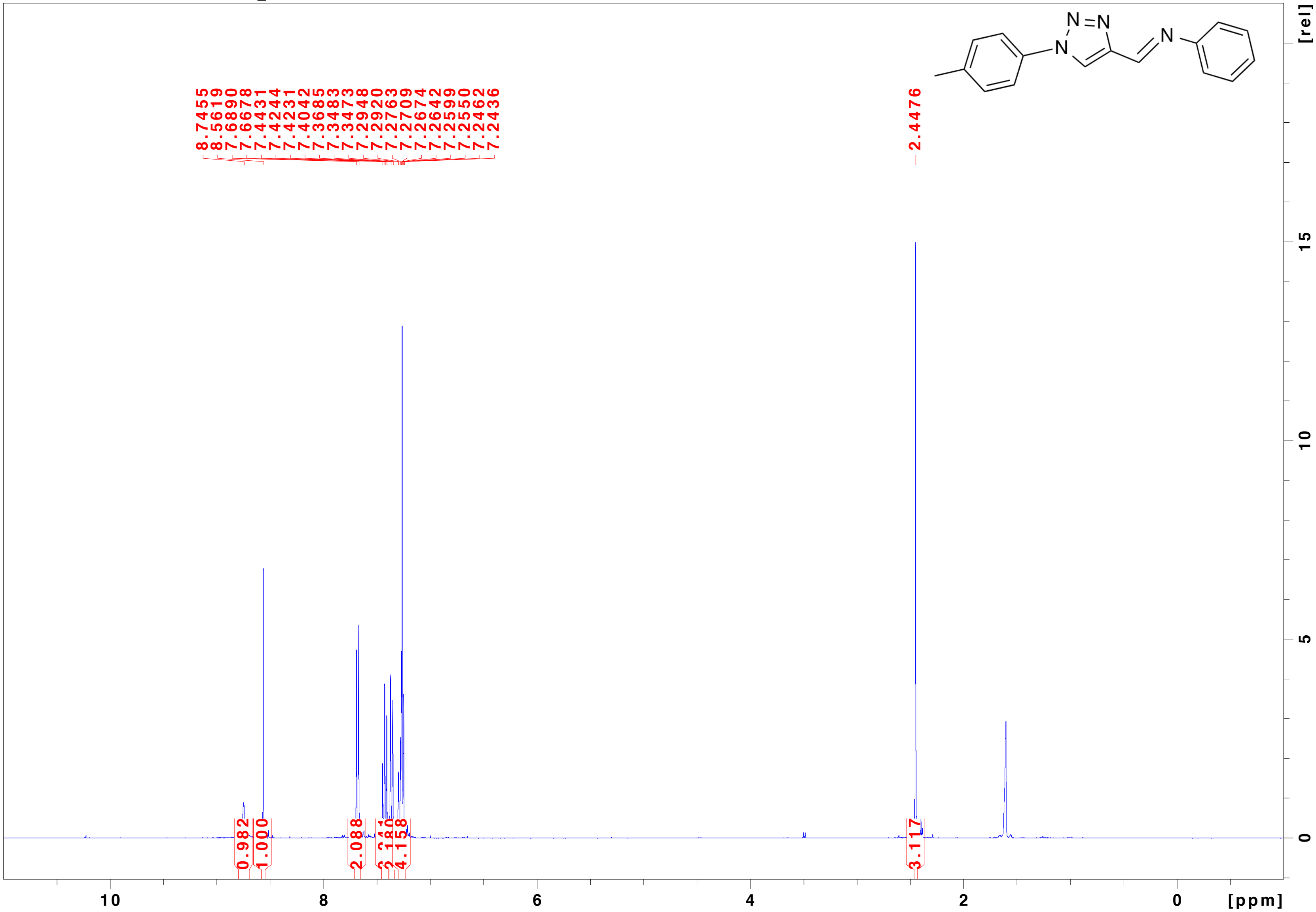

^{13}C NMR spectrum of **2dc'**

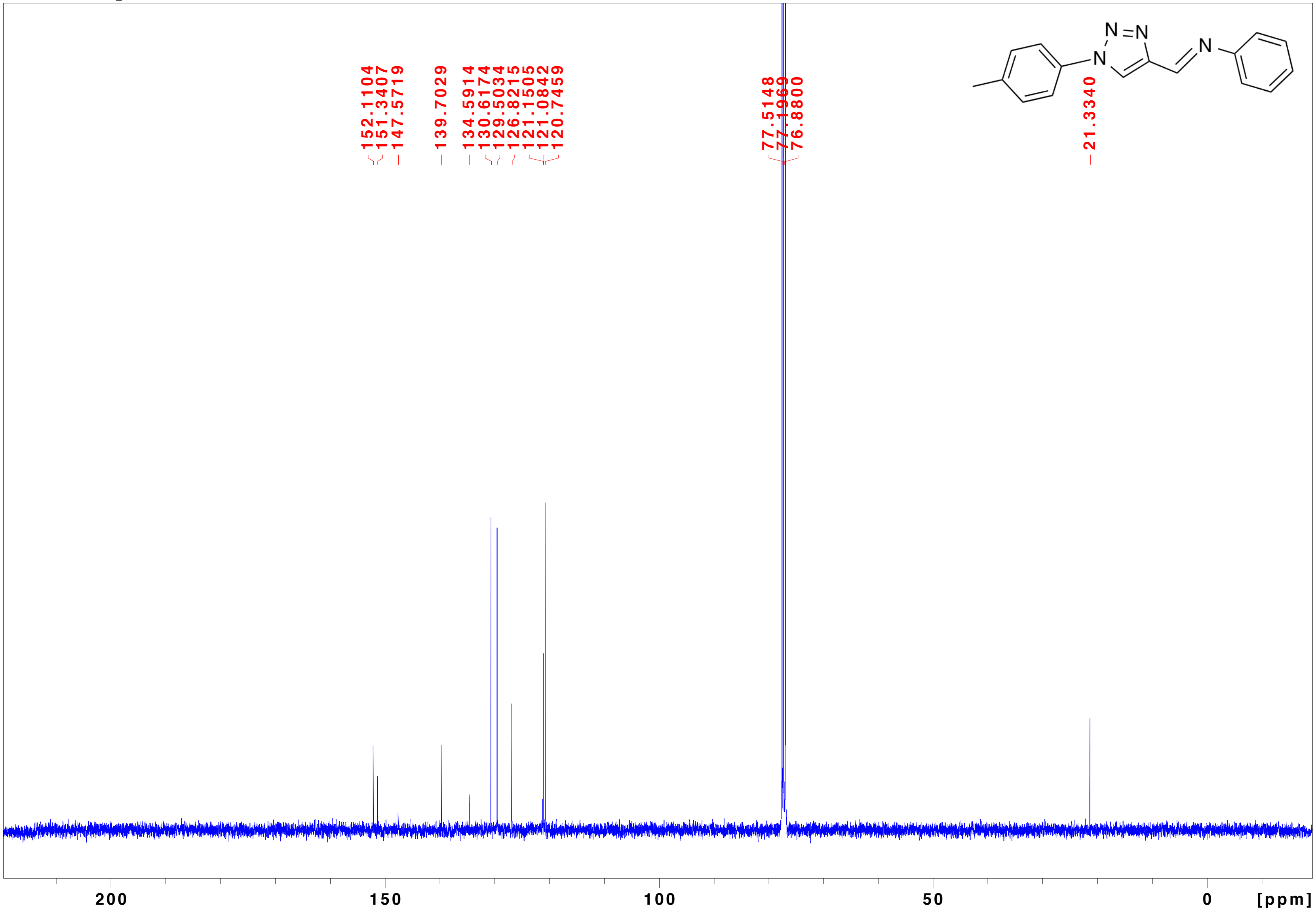

^{1}H NMR spectrum of **2ec'**

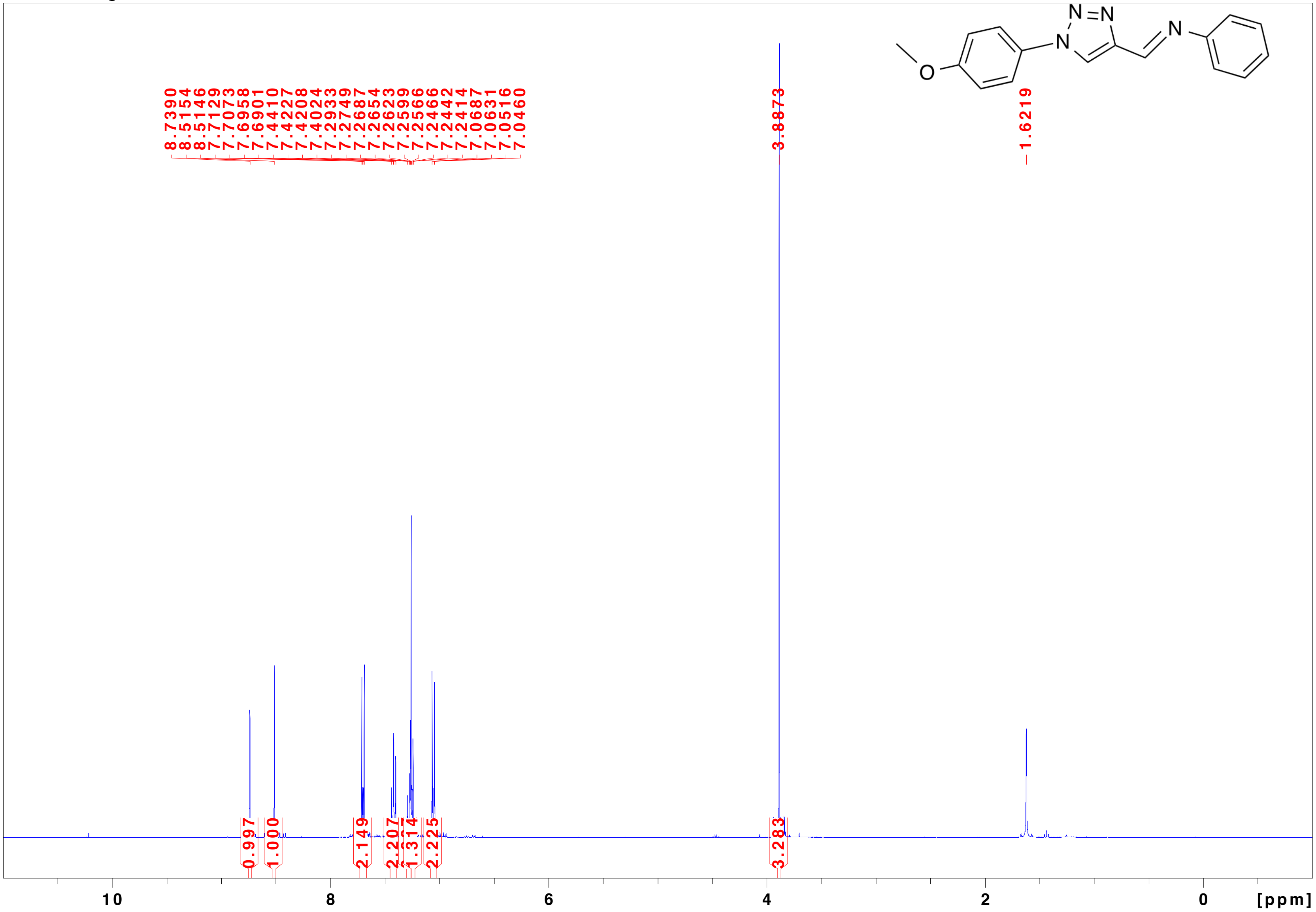

^{13}C NMR spectrum of **2ec'**

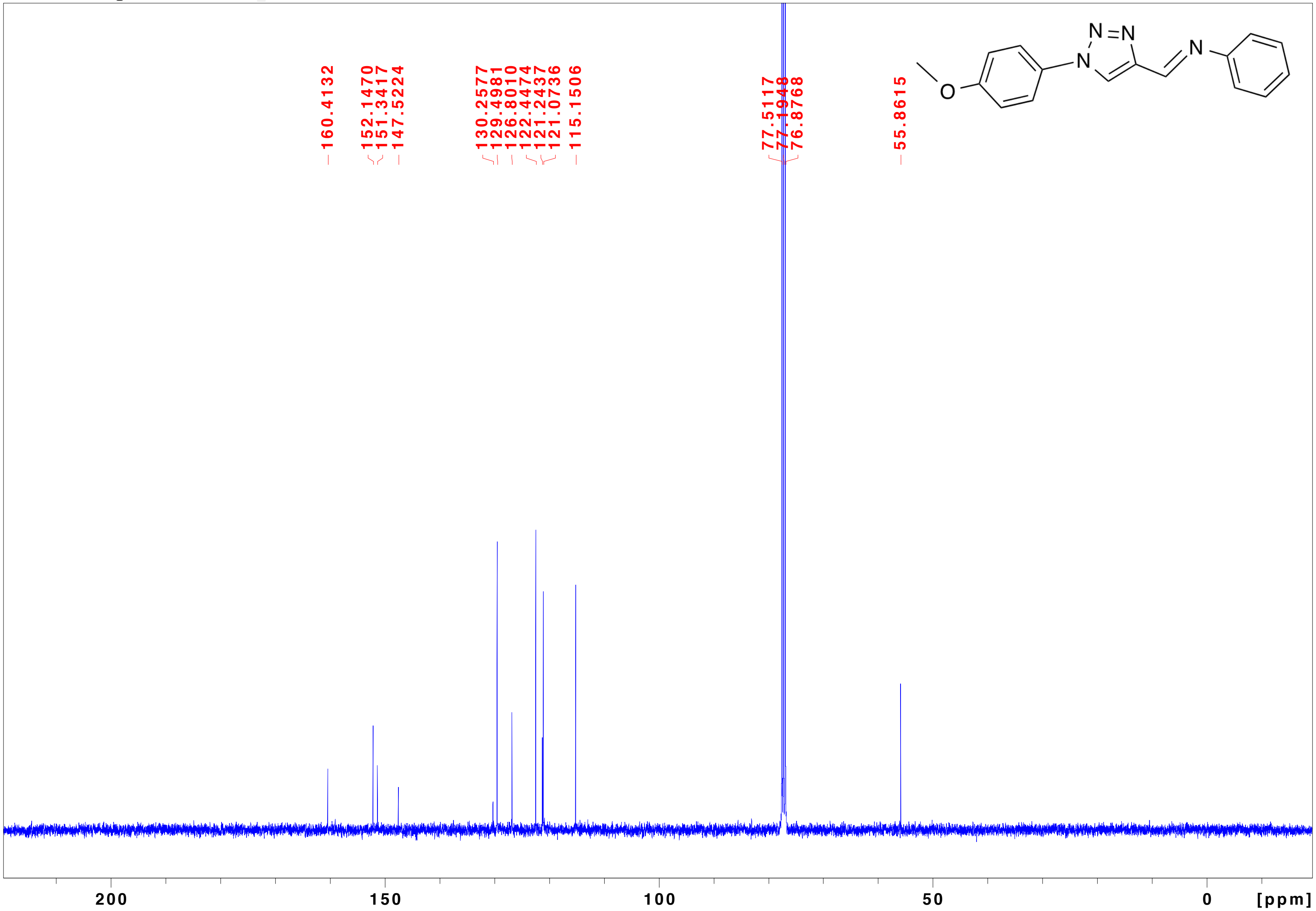

^{1}H NMR spectrum of **2fc’**

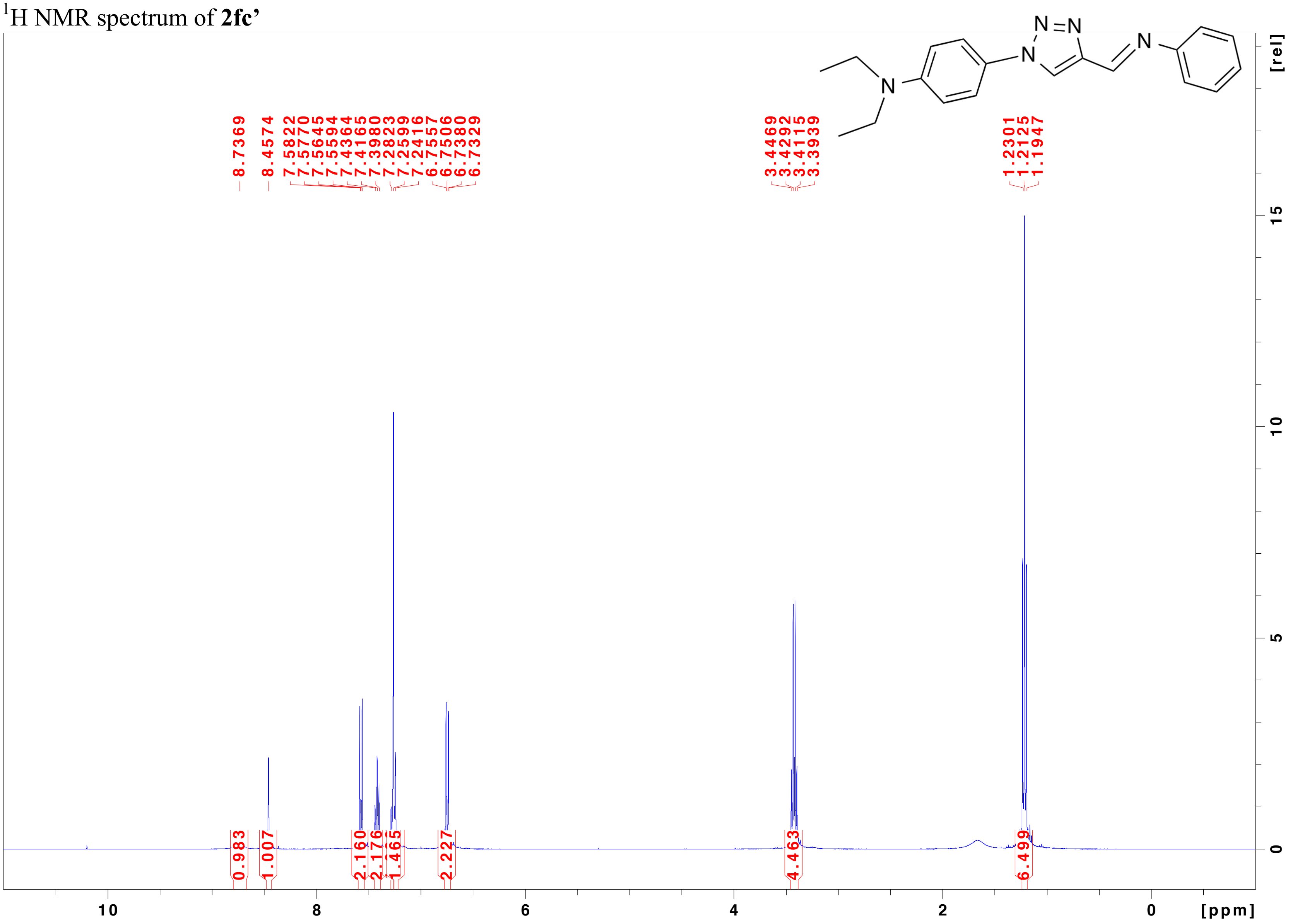

^{13}C NMR spectrum of **2fc'**

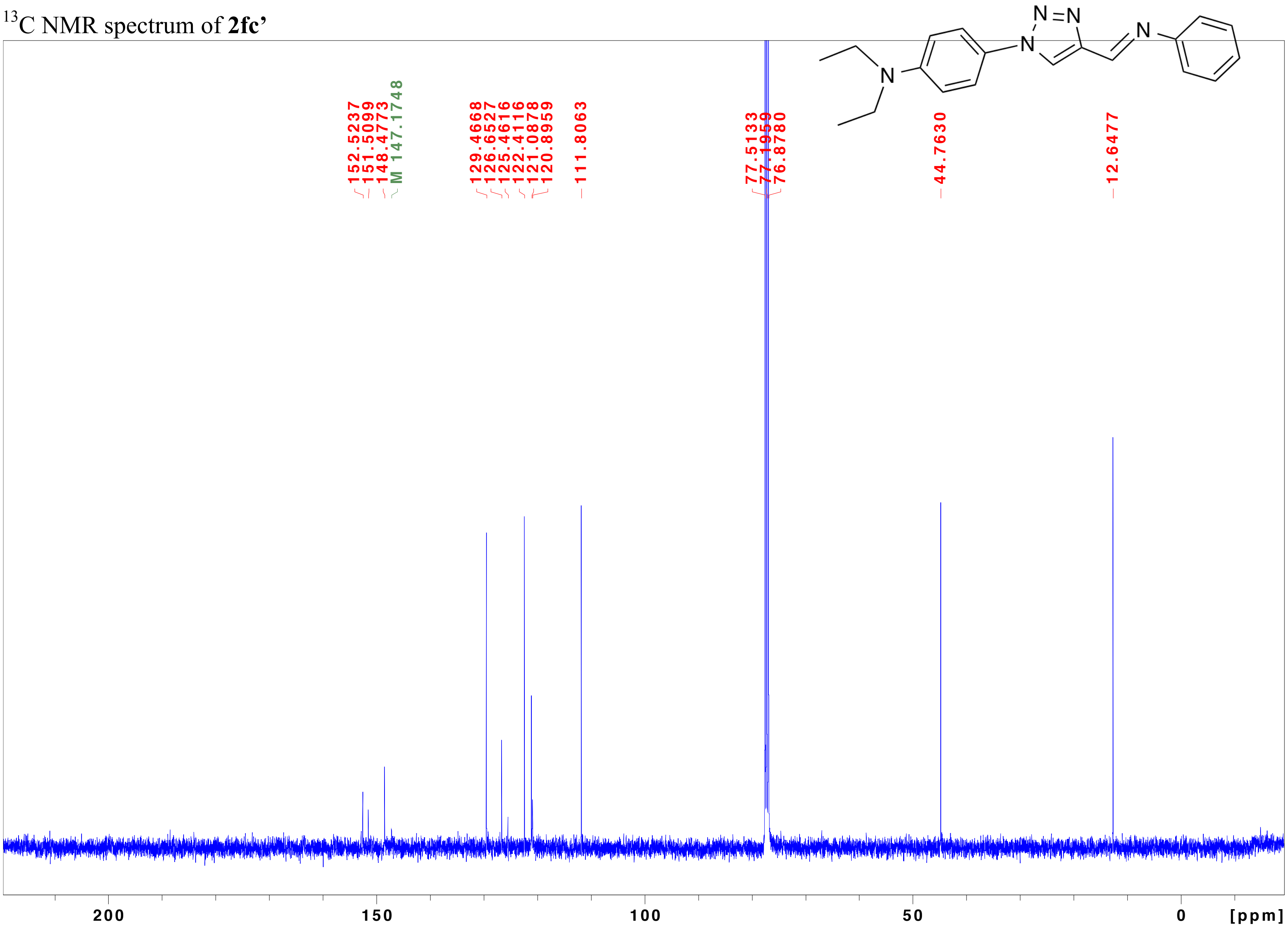

^{1}H NMR spectrum of **2cd’**

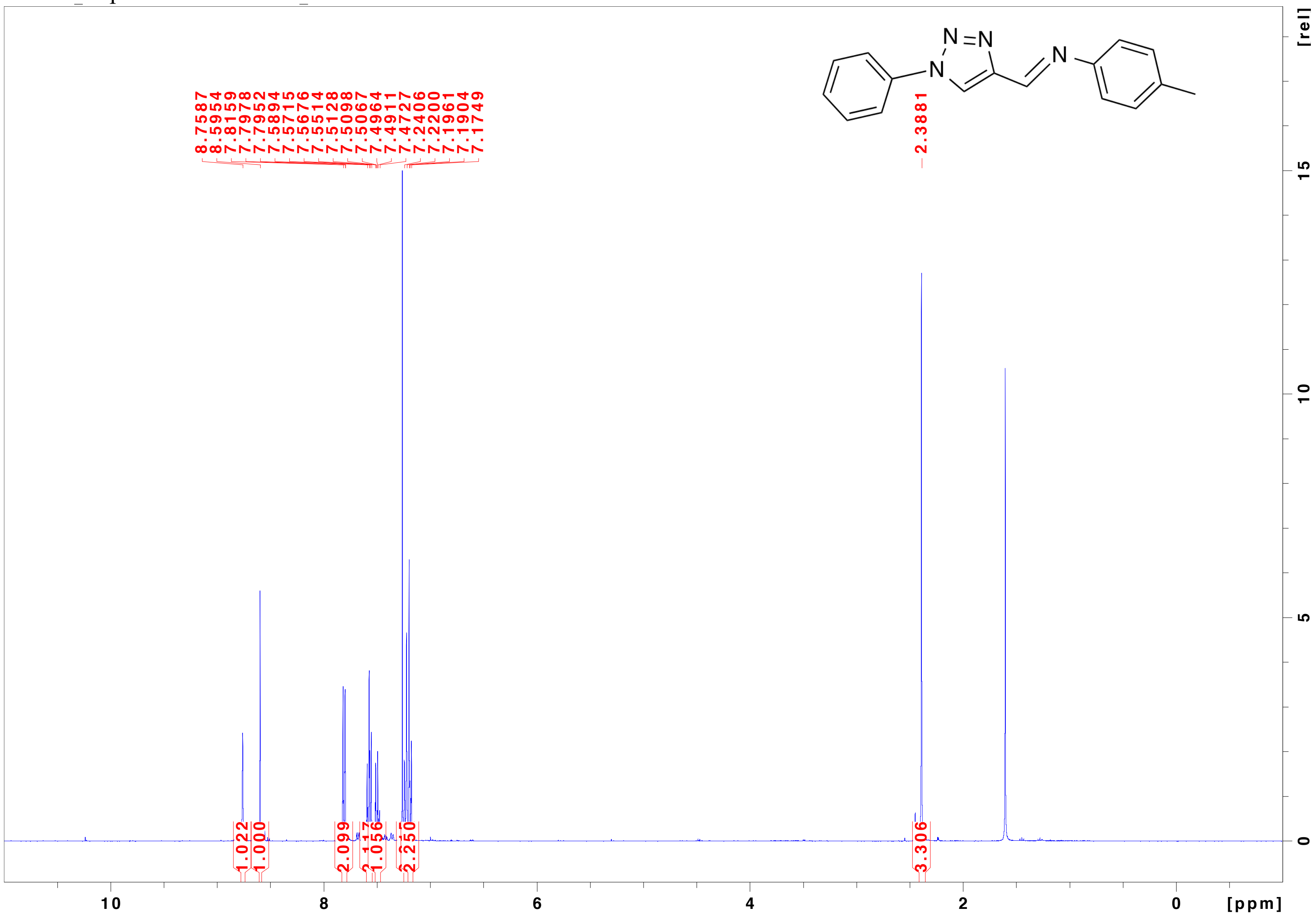

^{13}C NMR spectrum of **2cd'**

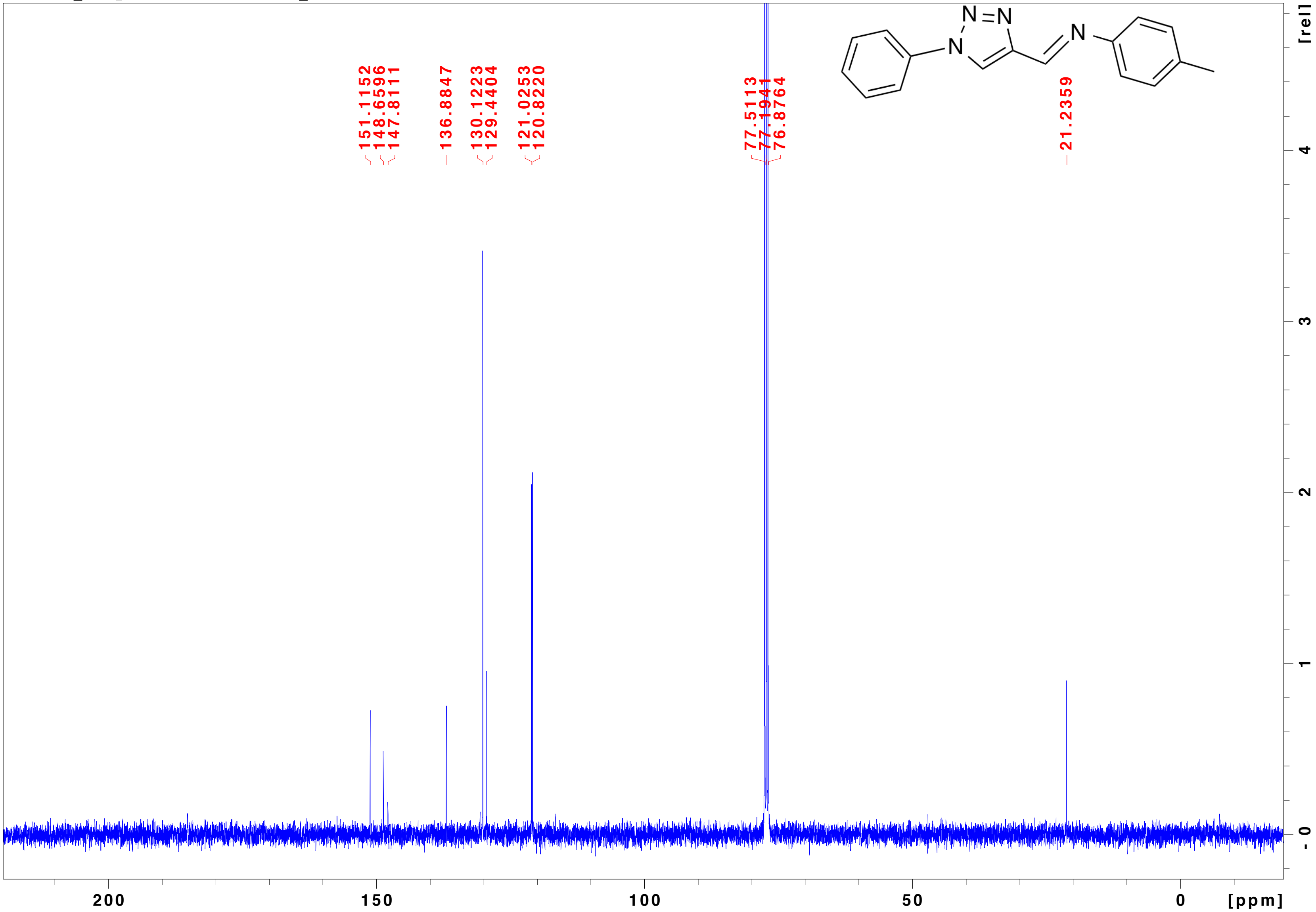

^{1}H NMR spectrum of **2ce'**

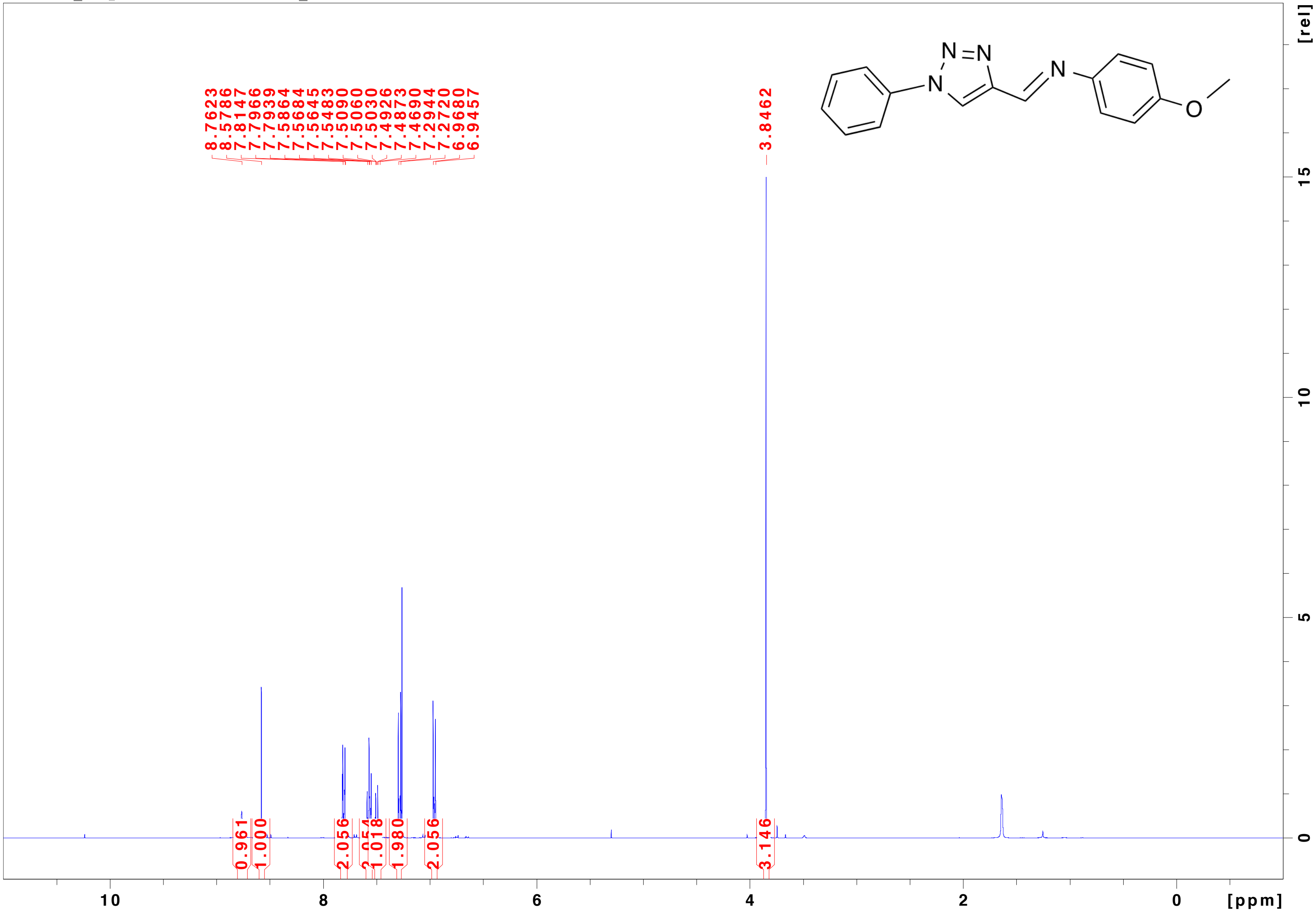

^{13}C NMR spectrum of **2ce'**

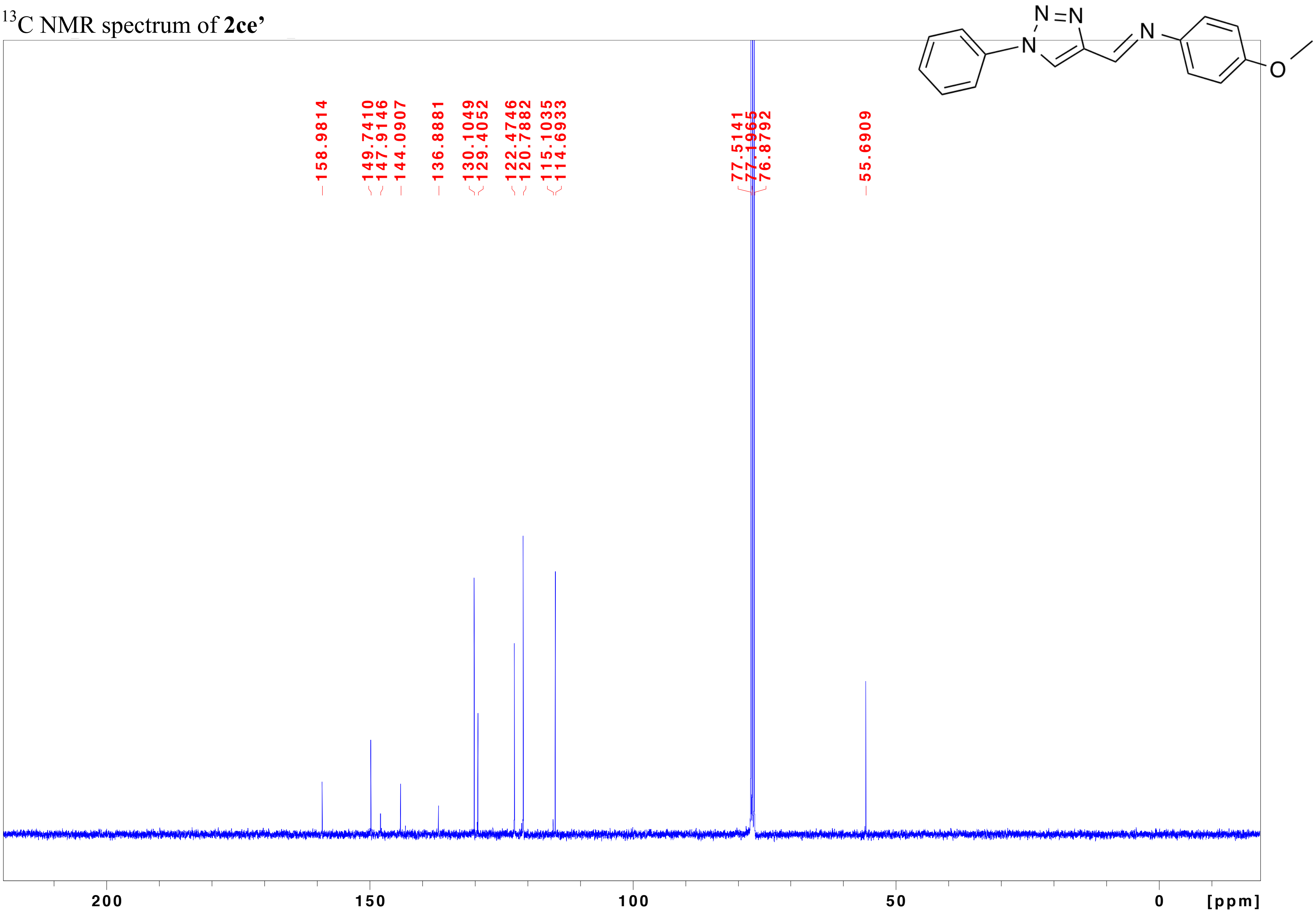

Examples of UV-Vis Kinetic Monitoring at 385 nm

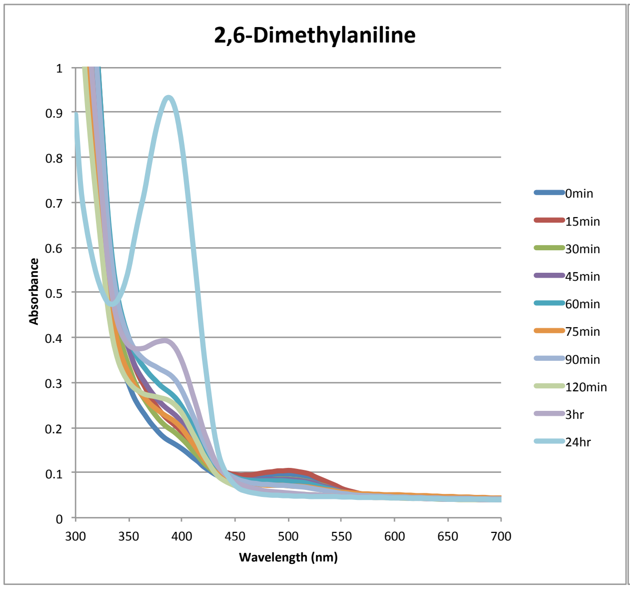

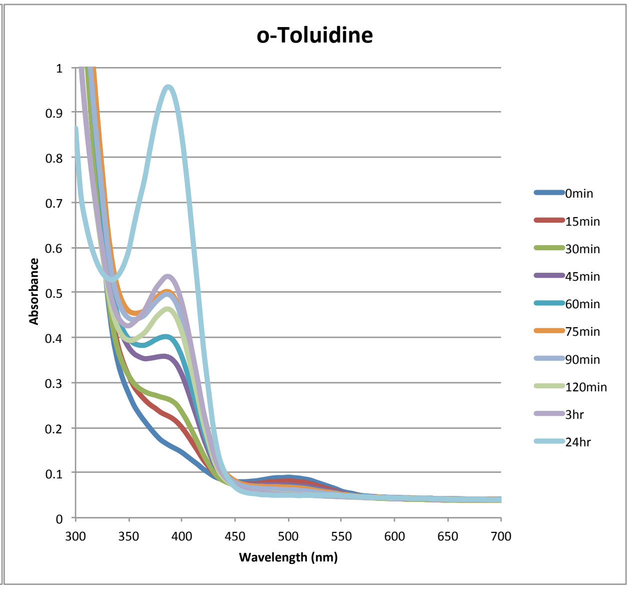

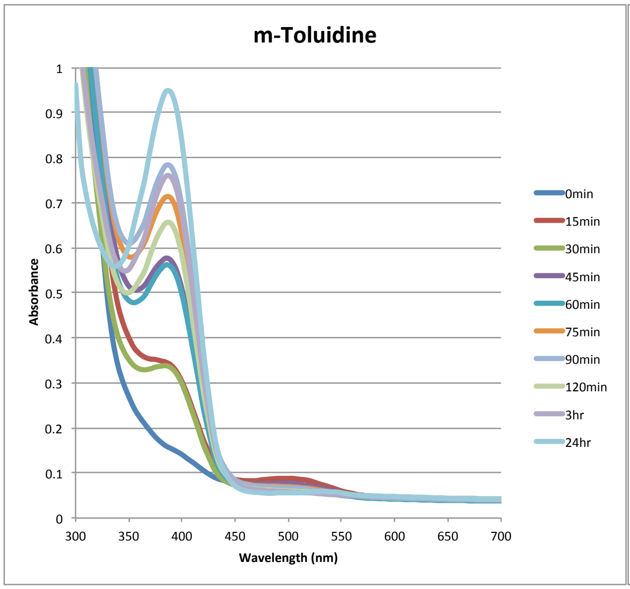

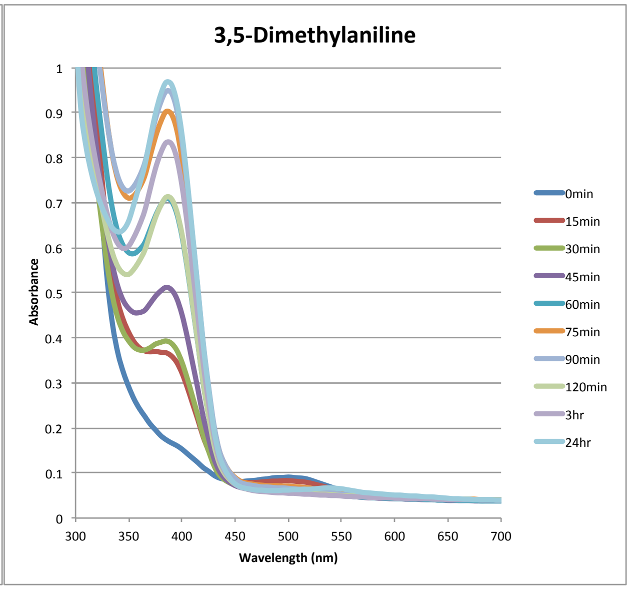

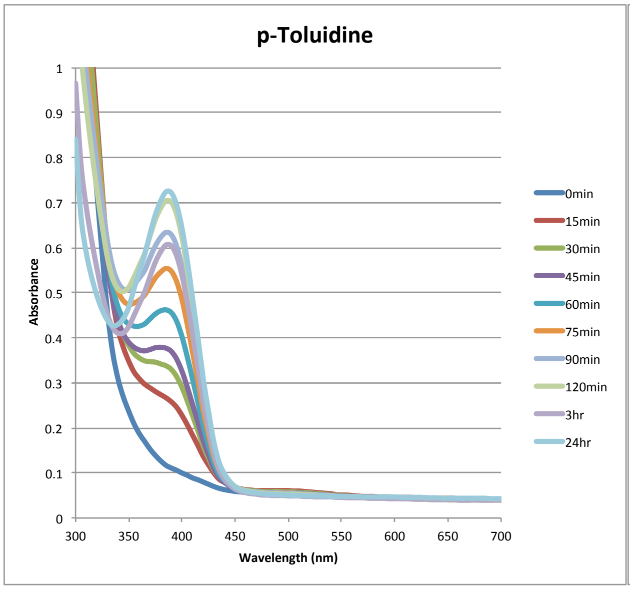
p-Toluidine
Absorbance
Wavelength (nm)
0min
15min
30min
45min
60min
75min
90min
120min
3hr
24hr

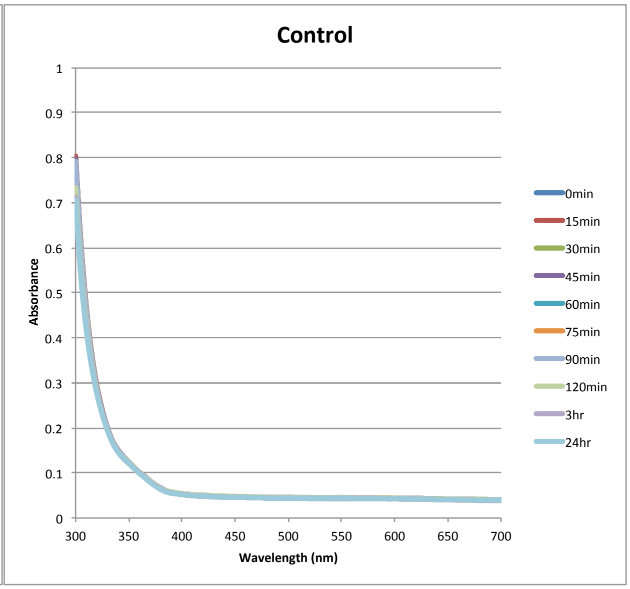
Control
Absorbance
Wavelength (nm)
0min
15min
30min
45min
60min
75min
90min
120min
3hr
24hr

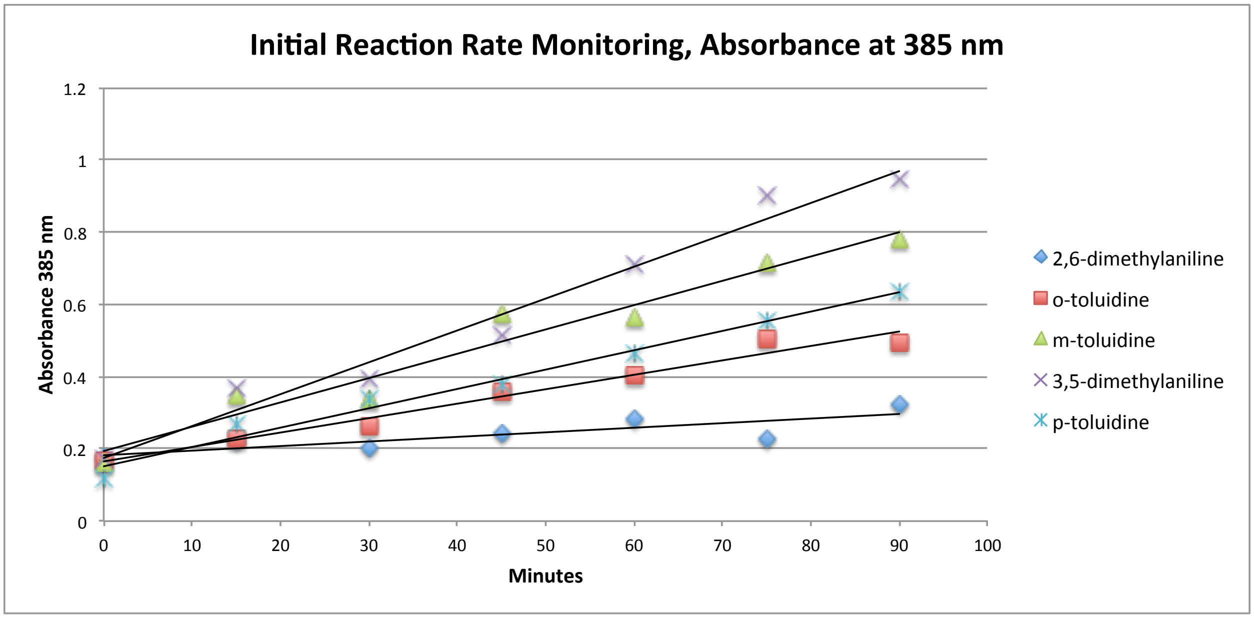
Initial Reaction Rate Monitoring, Absorbance at 385 nm
Absorbance 385 nm
Minutes
2,6-dimethylaniline
o-toluidine
m-toluidine
3,5-dimethylaniline
p-toluidine
